# Study on the performance and mechanism of a p–n type In_2_O_3_/BiOCl heterojunction prepared using a sacrificial MOF framework for the degradation of PFOA†

**DOI:** 10.1039/d5ra01317h

**Published:** 2025-05-08

**Authors:** Zhen Hu, He Li, Hailian Yu

**Affiliations:** a a, School of Chemical Engineering, Sichuan University of Science & Engineering Sichuan P. R. China huz88888@126.com

## Abstract

In this study, an In_2_O_3_/BiOCl p–n heterojunction was prepared using a co-calcination method. By utilising the built-in electric field formed near the heterojunction interface, photoinduced electron–hole pairs can be effectively separated, thereby enhancing the photocatalytic activity of the photocatalyst. Experimental results indicate that the p–n heterojunction photocatalyst significantly enhanced photocatalytic activity in the degradation of PFOA under UV light irradiation. Within 2 h, the defluorination rate of PFOA achieved by the heterojunction photocatalyst reached 84.01%, while the pure BiOCl and In_2_O_3_ photocatalysts exhibit defluorination rates of 61.82% and 56.69%, respectively. The degradation mechanism of PFOA was studied through free radical capture experiments, VB-XPS, FT-IR, and LC-MS. Mechanistic studies show that the main active substances in the heterojunction are holes (h^+^) and superoxide radicals (˙O_2_^−^). The holes in the valence band of In_2_O_3_ are transferred to BiOCl under the effect of the built-in electric field, and the defluorination of PFOA mainly occurs on the BiOCl component of the heterojunction. This highlights the superiority of heterojunctions over pure photocatalysts in terms of their photocatalytic efficiency and provides insights into the photocatalytic degradation mechanism of PFOA.

## Abstract 2

In this study, an In_2_O_3_/BiOCl p–n heterojunction was prepared using a co-calcination method.

## Graphical abstract

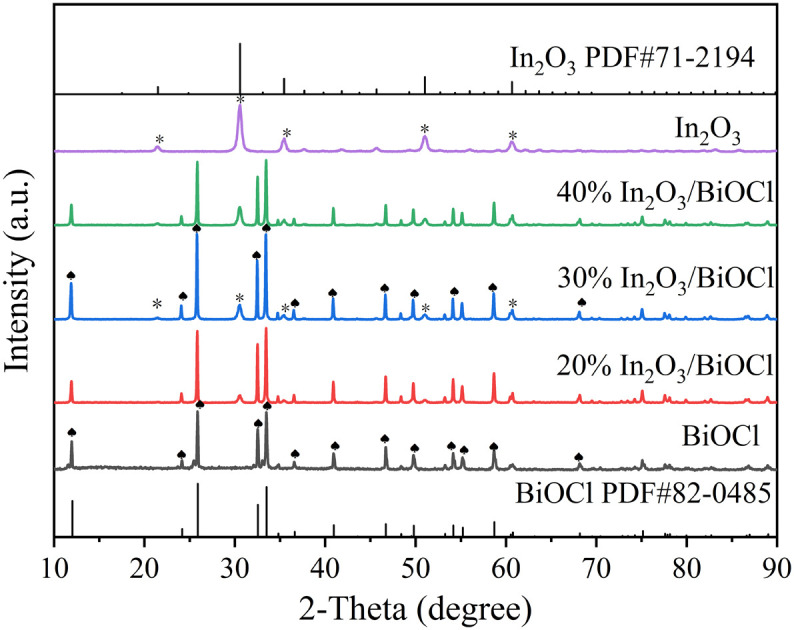

## 1. Introduction

Perfluorooctanoic acid (PFOA) is a type of synthetic perfluorinated organic compound that can be almost completely ionized in water. As a strong organic acid, it can corrode the skin and has a molecular weight of 414.06 g mol^−1^.^1^ Since 1950, Minnesota Mining and Manufacturing Company (3M) has been developing perfluorinated compounds, which have been widely used in various fields, such as refrigerants, polymers, flame retardants, surfactants, and pharmaceuticals.^2^ However, PFOA is commonly detected in groundwater, soil, and biota owing to its persistent and bioaccumulative nature.^3,4^ Moreover, some studies have found a close relationship between PFOA and human health, including increased cholesterol and liver enzyme levels, significantly increased incidence of kidney cancer and testicular cancer, reduced fertility, developmental effects on children's lungs, decreased immunity, and induction of thyroid disease.^5,6^ Some studies have shown that PFOA can have an impact on fetal development through maternal exposure.^7,8^ Therefore, it is urgent to develop efficient technology for eliminating PFOA pollutants in water using photocatalysis to protect human health.

In recent years, researchers worldwide have conducted numerous studies on the degradation of PFOA, including those based on reverse osmosis,^9^ adsorption,^10^ electrochemistry,^11^ advanced oxidation/reduction^12^ and photocatalytic degradation.^13–15^ Although adsorption and reverse osmosis methods can effectively remove PFOA from wastewater, further treatment is required for the removal of residual substances. In the processes of electrochemical and advanced oxidation degradation of PFOA, hydrogen atoms on the carbon chain of PFOA molecules are completely replaced by fluorine atoms. This results in the carbon atoms being surrounded by fluorine atoms in the spatial structure, making it impossible for external active groups to directly attack the carbon chain.^16^ Moreover, thermodynamically, owing to the high bond energy of the C–F bond (485 kJ mol^−1^),^17^ the C–F bond is very stable, resulting in the low efficiency of these two methods in PFOA degradation. Photocatalytic degradation technology has strong oxidative ability compared with the commonly used degradation technologies and exhibits particularly strong selectivity towards refractory organic compounds.^18^ Therefore, scholars worldwide are currently employing photocatalytic degradation techniques to study the degradation of PFOA. However, as shown in Table 1, most of the previous studies were time-consuming with low degradation rates, and the degree of mineralization of PFOA was not obvious. Thus, it is necessary to further develop a photocatalyst that can efficiently degrade PFOA.

**Table 1:** Photocatalysis technologies for PFOA degradation

Photocatalyst	Light wavelength	Conditions	Reaction time	Degradation & mineralization ratio	Ref.
BiOHP/CS	254 nm UV lamp	*C*_0_ = 0.12 mM	4 h	99% & 32.5%	19
		*C*_catalyst_ = 0.5 g L^−1^			
		pH = 5			
In-MOF/BiOF	254 nm UV lamp	*C*_0_ = 15 mg L^−1^	3 h	99% & 34%	20
		*C*_catalyst_ = 0.5 g L^−1^			
BiOI@Bi_5_O_7_	Solar irradiation	*C*_0_ = 15 mg L^−1^	6 h	80% & 60%	21
		*C*_catalyst_ = 0.5 g L^−1^			
		pH = 3			
BiOF	UV lamp	*C*_0_ = 15 mg L^−1^	6 h	99% & 26%	22
		*C*_catalyst_ = 0.5 g L^−1^			
ZIF67/MIL-100(Fe)@C_3_N_4_	Iodine–tungsten lamp	*C*_0_ = 10 mg L^−1^	8 h	79.2% & —	23
		*C*_catalyst_ = 1 g L^−1^			
		pH = 4.6			
BiOCl	UV lamp	*C*_0_ = 20 mg L^−1^	4 h	— & 29.93%	24
		*C*_catalyst_ = 1 g L^−1^			
		pH = 3.8			
In_2_O_3_/BiOCl	UV lamp	*C*_0_ = 20 mg L^−1^	2 h	— & 84.01%	This work
		*C*_catalyst_ = 0.2 g L^−1^			
		pH = 5.0			

BiOCl is a common p-type semiconductor material.^25^ Due to its unique layered structure, its electronic transition is an indirect bandgap transition. The [Bi_2_O_2_] atom-staggered layered structure provides enough space to polarize atoms and orbitals,^26,27^ thereby forming internal electric fields perpendicular to each layer, and effectively separating the generated electron–hole pairs. It has been reported that BiOCl exhibits exceptional degradation performance for the photocatalytic degradation of PFOA.^28–30^ However, during its degradation process, the generated short-chain perfluoro carboxylic acids (PFCAs) resulting from the incomplete mineralization of PFOA still can pose a threat to the ecological environment,^31^ so further research is needed to achieve complete defluorination and mineralization. The construction of heterojunctions can effectively improve the photocatalytic performance of semiconductors.^32^ The reason is that in a heterojunction, the establishment of the heterojunction interface enhances the transfer of photo-generated charges, thereby enhancing the photocatalytic activity of the heterojunction.

In_2_O_3_ is an n-type semiconductor that is commonly used in photocatalytic oxidation studies due to its narrow bandgap (2.7 eV) and simple, low-cost preparation method.^33^ However, the photocatalytic efficiency of In_2_O_3_ is low due to the easy recombination of its photo-generated electron–hole pairs. To enhance the photocatalytic efficiency of In_2_O_3_, it can be combined with other p-type semiconductors to form a p–n heterojunction. An internal electric field will be generated near the interface of the heterojunction, which will guide the photogenerated electrons produced in the heterojunction to transfer a conduction band (CB) in the n-type semiconductor,^25^ while the photogenerated holes will remain in the valence band (VB) of the p-type semiconductor. Therefore, the internal electric field near the interface will improve the separation efficiency and activity of the photogenerated electron–hole pairs.

In this study, an In_2_O_3_/BiOCl composite material was prepared by a simple mixing-calcination method. The formation of a p–n heterojunction for the composite material In_2_O_3_/BiOCl significantly improved the photocatalytic degradation of PFOA. The photocatalytic performance of the prepared p–n heterojunction In_2_O_3_/BiOCl was systematically investigated by defluorination mineralization of refractory organic pollutants PFOA under ultraviolet light. The roles of various active species in the photocatalytic degradation process were identified, and the pathways of electron transfer, photocatalytic degradation mechanism, and degradation pathway of PFOA in the p–n heterojunction were revealed.

## 2. Experimental

### 2.1 Chemical reagents

Perfluorooctanoic acid (PFOA, Shanghai Adamas Reagent Co., Ltd, 96.0%), hydrated indium(iii) nitrate (In(NO_3_)_3_·*x*H_2_O, Adamas, 99.9%), *p*-phthalic acid (C_8_H_6_O_4_, Adamas, 99.0%) sodium chloride (NaCl, Chengdu Cologne Chemical Co., Ltd, 99.5%), acetic acid (CH_3_COOH, Adamas, 99.0%), potassium bromate (KBrO_3_, Cologne, 99.8%), bismuth nitrate hydrate (Bi(NO_3_)_3_·5H_2_O, Cologne, 99.0%), methanol (CH_3_OH, Cologne, 99.5%), ascorbic acid (C_6_H_8_O_6_, Cologne, 99.7%), ethylenediaminetetraacetic acid disodium salt (C_10_H_14_N_2_Na_2_O_8_, Cologne, 99.0%), sodium hydroxide (NaOH, Cologne, 98.0%), ethanol (C_2_H_5_OH, Cologne, 99.7%) and nitric acid (HNO_3_, East Sichuan Chemical Group Co., Ltd, 65.0–68.0%). Deionized water was used in all experiments.

### 2.2 Preparation of photocatalysts

#### 2.2.1 Preparation of BiOCl

The BiOCl photocatalyst was prepared by hydrothermal method using bismuth nitrate and NaCl as raw materials. 0.01 mol Bi(NO_3_)_3_·5H_2_O and 0.01 mol NaCl were dissolved in glacial acetic acid and deionized water, respectively, and then an appropriate amount of methanol was added to an acetic acid solution containing bismuth nitrate. NaCl solution was added dropwise to the above solution, and the mixture was stirred for 0.5 h, transferred to a polytetrafluoroethylene reactor, and reacted at 160 °C for 24 h. The above reaction products were cooled to room temperature, filtered, and washed three times with deionized water and ethanol, respectively. The obtained solid product was dried at 80 °C for 12 h, and the final sample was obtained.

#### 2.2.2 Preparation of In_2_O_3_

First, 0.60 g of In(NO_3_)_3_·*x*H_2_O (0.2 mol) and 0.33 g of PTA were dissolved in 50 mL *N*,*N*-dimethylformamide solution, and then irradiated for 20 min under ultrasonication. The reaction solution was transferred to a reaction kettle lined with polytetrafluoroethylene, and maintained at 130 °C for 4 h. After cooling to room temperature and filtering, the product was washed with ethanol three times, and then dried in a vacuum drying oven at 50 °C for 12 h to obtain the precursor. The precursor was thoroughly ground and transferred to a crucible, and calcined at 550 °C in a muffle furnace at a heating rate of 3 °C per min for 1 h. The final product, In_2_O_3_, was obtained by cooling down to room temperature at a rate of 5 °C per min.

#### 2.2.3 Preparation of the In_2_O_3_/BiOCl heterojunction

The prepared In_2_O_3_ and BiOCl were weighed according to the mass ratio. The two materials were dispersed in 5 mL of EtOH, and then the mixture was sonicated for 20 min. The resulting suspension was dried at 60 °C for 12 h until completely dry. The reaction product was ground thoroughly and then transferred into an alumina crucible. The crucible was heated at a heating rate of 5 °C per min to 300 °C, and calcined for 3 h. The final product was named according to the different mass ratios, such as 20% In_2_O_3_/BiOCl, 25% In_2_O_3_/BiOCl, 30% In_2_O_3_/BiOCl, 35% In_2_O_3_/BiOCl, and 40% In_2_O_3_/BiOCl.

### 2.3 Characterization

The XRD pattern of the catalyst was determined using a D2 Phaser X-ray diffractometer (XRD, Cu Kα = 1.5406 Å, Bruker). The specific surface area (*S*_BET_) of the samples was measured by SSA-4200 (Builder, Beijing). The morphology of the catalyst was analyzed by scanning electron microscopy (SEM, Tescan Vega 3 SBU). X-ray photoelectron spectroscopy (XPS) (Escalab 250Xi, Thermo Fisher Scientific) was used to investigate the surface chemical composition of the heterojunction. The diffuse reflectance spectroscopy (DRS) of the catalyst was recorded using a UV-visible spectrophotometer (UV-2550, Shimadzu). The e^−^/h^+^ recombination rate was tested on a fluorescence spectrometer (PL, PICOQuant FT-300). Electrochemical impedance spectroscopy (EIS) and transient photocurrent (*I*–*t*) tests were performed on an electrochemical workstation (CHI660E). An ion chromatography (Essentia IC16, Shimadzu) system coupled with an ion meter (PXSJ-216F) was employed to quantify the fluoride ion (F^−^) concentrations during the experimental analysis. A TOC analyzer (TOC-L, Shimadzu) was used to determine the removal efficiency of organic carbon in PFOA. The electron spin resonance spectrometer (ESR, Bruker MX-PLUS) was used to test the free radicals produced by the samples. The adsorption of the catalyst for PFOA was tested by Fourier transform infrared spectrometer (FT-IR, Bruker).

The detection and analysis of the intermediate products generated during the degradation of PFOA were carried out using high-performance liquid chromatography/quadrupole time-of-flight mass spectrometry (HPLC/QTOF/MS). MassLynx V4.1 was used for data analysis. The specific testing parameters included an ACQUITY UPLC BEH C18 column (1.7 μm, 2.1 mm × 50 mm) with a column temperature of 40 °C and injection volume of 3 μL. An acetic acid ammonium solution (2.5 mmol L^−1^) was used as mobile phase A, while acetonitrile was used as mobile phase B for elution. In the gradient elution mode, mobile phase A accounts for 90–58%, 58–30%, 30–25%, and 25–90% of the total eluent volume at 0–3 min, 3–5 min, 5–8 min, and 8–12 min, respectively. The flow rate is set at 0.4 mL per minute. Mass spectrometry employs electrospray ionization (ESI) as an ion source, utilizing the negative ion mode and leucine enkephalin as an online calibration substance. The cone voltage is set at 40 V, with the collision energy ranging from 20–45 V for LC-MS. The ion source temperature is set at 100 °C and the desolvation temperature at 300 °C. The capillary voltage is set at 3 kV, and the desolvation gas flow rate is 600 L per h. Data acquisition is acquired through the MSE mode.

### 2.4 Photocatalytic defluorination of PFOA

To achieve enhanced degradation of PFOA, various light sources were systematically evaluated, with ultraviolet (UV) irradiation being ultimately selected as the optimal condition for PFOA decomposition. UV light irradiation was provided by a 500 W mercury lamp. In order to reduce the heat generated by the lamp during the degradation of PFOA, the lamp was placed in a cylindrical quartz water jacket and completely wrapped by a quartz water jacket. The degradation temperature was maintained by cooling water. Before the mercury lamp was turned on, 50 mL PFOA solution was prepared and stirred by magnetic stirring in the dark for half an hour to reach adsorption–desorption equilibrium. At fixed intervals throughout the degradation process, equal samples of degradation solution were taken out. To investigate the effect of the preparation conditions on heterojunctions, the degradation effect of heterojunctions prepared under different conditions was tested under the same conditions. The influence of factors such as the dosage of the photocatalyst, pH, and initial concentration of PFOA on the degradation of PFOA was investigated. The experiments for capturing the reactive species were similar to the previous photocatalytic activity tests. Different scavengers, including ethylenediaminetetraacetic acid disodium salt (EDTA), benzoquinone (BQ), and isopropanol (IPA), were added to the PFOA solution to capture holes (h^+^), superoxide radicals (˙O_2_^−^), and hydroxyl radicals (OH^−^).

## 3. Results and discussion

### 3.1 Characterization and analysis of In_2_O_3_/BiOCl heterojunction

**Fig. 1:**
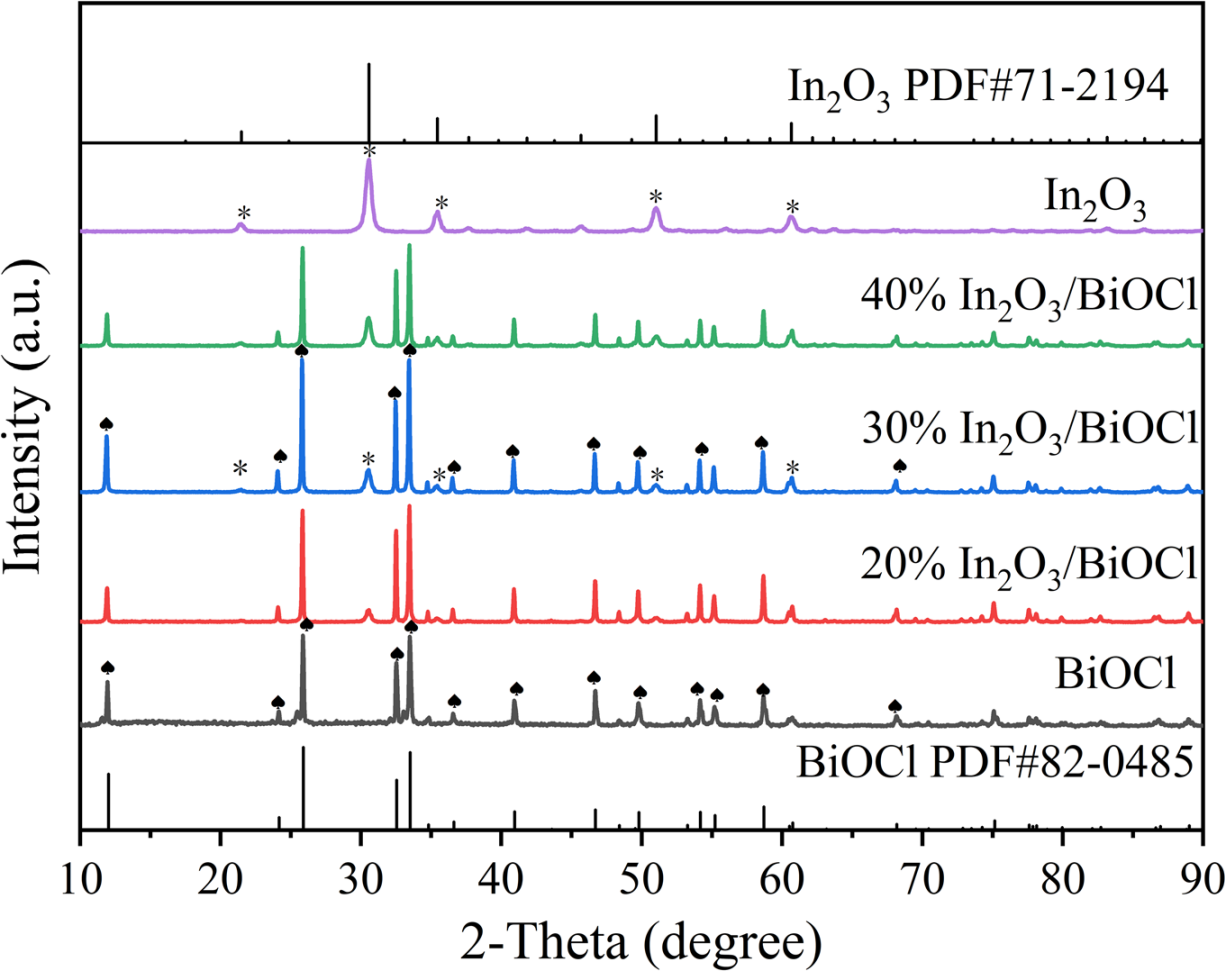
XRD spectra of In_2_O_3_, BiOCl, and the In_2_O_3_/BiOCl heterojunction with different mass ratios.

The XRD pattern of the prepared photocatalyst is shown in Fig. 1. The diffraction peaks at 2*θ* = 21.5 (211), 30.6 (222), 35.5 (400), 51.0 (440), and 60.7 (622) in the In_2_O_3_ sample spectrum are in good agreement with the standard pattern of the cubic phase In_2_O_3_ (PDF #71-2194).^34^ In the BiOCl sample spectrum, all diffraction peaks are similar to those of the diffraction peaks of the pure tetragonal phase structure BiOCl standard (PDF #82-0485).^35^ The XRD spectra of the pure In_2_O_3_ and BiOCl samples do not exhibit any impurity peaks, indicating the high purity of the samples.^36^ However, it can be observed that the diffraction peaks of the prepared In_2_O_3_ are broad and weak, indicating that the crystallinity of the sample is slightly inferior to that of BiOCl. The XRD spectra of the In_2_O_3_/BiOCl photocatalysts with different composite ratios show the characteristic diffraction peaks of In_2_O_3_ and BiOCl (marked in Fig. 1), indicating the successful preparation of the In_2_O_3_/BiOCl heterojunction. Furthermore, according to the changing trend of peaks in the X-ray diffraction (XRD) spectra, it can be observed that as the proportion of In_2_O_3_ in the composite material increases, the characteristic peak of In_2_O_3_ gradually becomes stronger.

**Fig. 2:**
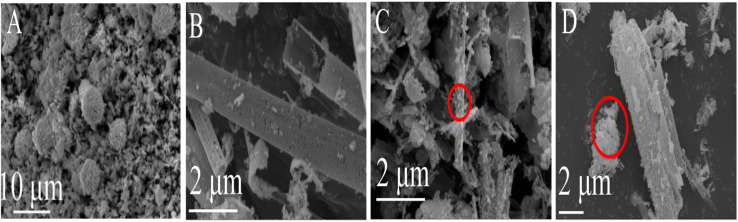
SEM images of the photocatalyst. (A) BiOCl; (B) In_2_O_3_; (C and D) 30% In_2_O_3_/BiOCl.

The morphology of the prepared photocatalyst was observed using SEM to reveal its microstructure. The morphologies of BiOCl, In_2_O_3_, and 30% In_2_O_3_/BiOCl are shown in Fig. 2. SEM images of BiOCl and In_2_O_3_ are presented in Fig. 2(A) and (B), respectively. BiOCl is represented in the form of small flakes and round-ball aggregates. Meanwhile, In_2_O_3_ exhibits an irregular shape of hollow rods with uneven sizes and a smooth surface, and some small particles adhere to its surface, which might cause detachment during calcination. SEM images of the 30% In_2_O_3_/BiOCl composite material, shown in Fig. 2(C and D), reveal that the combination between BiOCl and the In_2_O_3_ materials was mediated by the small flake-shaped BiOCl coating on the surface of the hollow rod-shaped In_2_O_3_ (Fig. 2(C)) or by particle-shaped In_2_O_3_ adhering to BiOCl that has fractured (Fig. 2(D)).

**Fig. 3:**
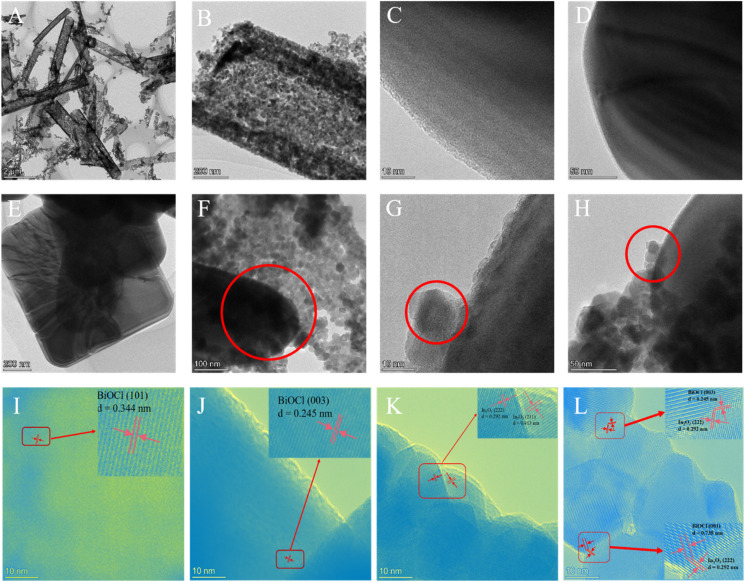
TEM (A–H) and HRTEM (I–L) images of the photocatalyst. (A, B, and K) In_2_O_3_; (C–E, I and J) BiOCl; (F–H and L) 30% In_2_O_3_/BiOCl.

In order to understand the microstructure of the catalyst, the prepared materials were probed using transmission electron microscopy (TEM). Fig. 3(A) and (E) show the TEM images of In_2_O_3_ and BiOCl, respectively. Fig. 3(A) further confirms the hollow rod-like morphology of In_2_O_3_. Fig. 3(F–G) presents the TEM images of the 30% In_2_O_3_/BiOCl composite material prepared in this study. Compared with Fig. 3(B) (In_2_O_3_) and Fig. 3(C and D) (BiOCl), it is confirmed again that the In_2_O_3_/BiOCl heterojunction was successfully constructed, indicating the proper combination of these two different materials in the heterojunction. The HRTEM images of BiOCl and In_2_O_3_ are displayed in Fig. 3(I, J and K). The lattice spacings of the (101) and (003) crystal planes of BiOCl, with values of 0.334 nm and 0.245 nm, respectively, are consistent with that reported in the literature for BiOCl (PDF #82-0485). The HRTEM image in Fig. 3(K) shows the lattice spacing of the (222) and (211) crystal planes of In_2_O_3_, with values of 0.292 nm and 0.413 nm, respectively, which is in good agreement with the standard values of In_2_O_3_ (PDF# 71-2194). Fig. 3(L) shows an HRTEM image of the In_2_O_3_/BiOCl heterostructure, where the (222) crystal plane of In_2_O_3_ intersects and overlaps with the (001) and (003) crystal planes of BiOCl, providing further evidence for the successful preparation of the In_2_O_3_/BiOCl heterostructure.

#### 3.1.1 BET specific surface area and pore size analysis of the photocatalyst

The specific surface area (*S*_BET_) and pore size of BiOCl, In_2_O_3_, and 30% In_2_O_3_/BiOCl were analyzed using N_2_ adsorption/desorption, and the results are presented in Table 2. It can be observed from this table that the specific surface area of BiOCl increases significantly from 3.42 m^2^ g^−1^ to 13.87 m^2^ g^−1^ after the introduction of In_2_O_3_. This increase in the specific surface area effectively enhances the adsorption of reactants on the catalytic surface, thereby promoting their absorption of light and enhancing the catalytic activity.

**Table 2:** Average crystallite size and specific surface area of samples

Sample	Specific surface area (m^2^ g^−1^)	Pore diameter (nm)	VP (mL g^−1^)
BiOCl	3.42	21.06	0.0182
30% In_2_O_3_/BiOCl	13.87	20.39	0.0707
In_2_O_3_	33.09	20.00	0.1655

**Fig. 4:**
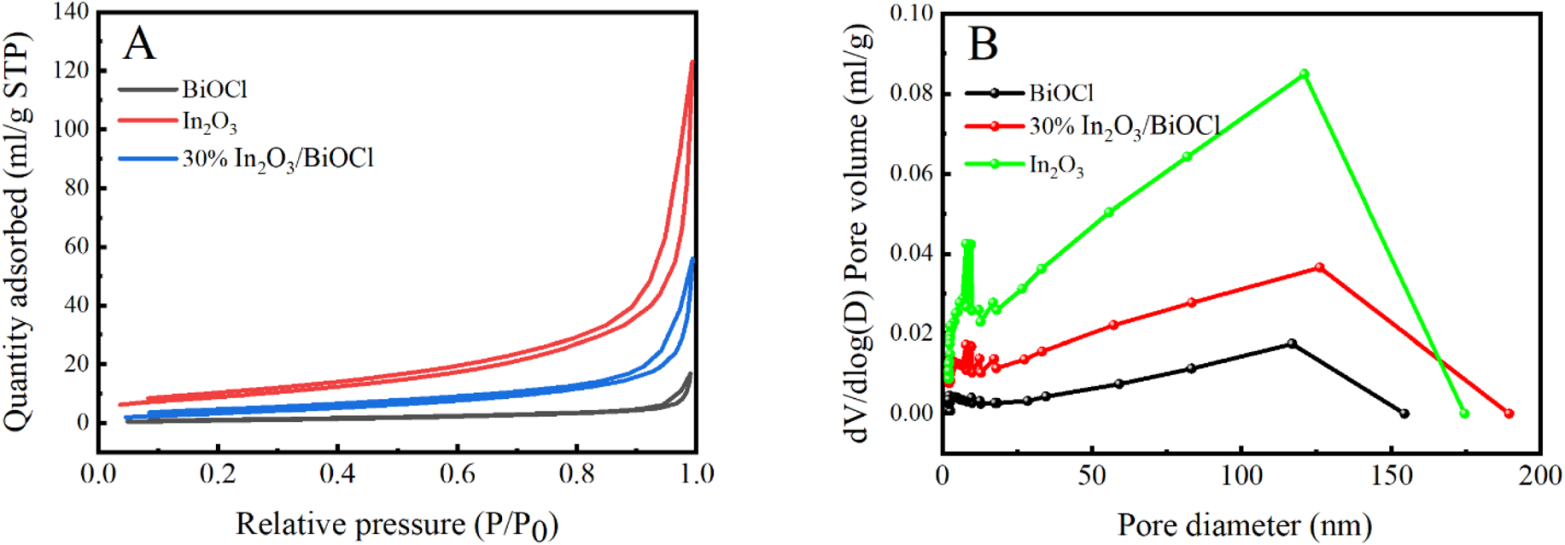
Nitrogen adsorption–desorption diagram of the photocatalyst (A), and micropore distribution diagram of the photocatalyst (B).

The nitrogen adsorption–desorption isotherms and pore size distribution curves of the prepared photocatalyst are shown in Fig. 4. It can be seen from Fig. 4(A) that the N_2_ adsorption–desorption isotherms of the three prepared samples match the trend of the type IV isotherm. Moreover, when the ratio of *P*/*P*_0_ is between 0.6 and 1.0, the hysteresis loops of the H3 adsorption can be observed according to the different sizes classified by IUPAC. So, it can be concluded that all three samples have spaces of different pore sizes. As shown in Fig. 4(B), the pore size of the prepared BiOCl, In_2_O_3_, and 30% In_2_O_3_/BiOCl are mainly distributed in the range of 50–150 nm. By comparison, the pore volume of BiOCl is the smallest, while the presence of the large pore volume in In_2_O_3_ and the heterojunction is conducive to the diffusion of photogenerated holes, which can effectively improve its photocatalytic activity. Compared with the BET specific surface area (33.09 m^2^ g^−1^) and pore volume (0.1655 mL g^−1^) of In_2_O_3_, the BET-specific surface area (13.87 m^2^ g^−1^) and pore volume (0.0707 mL g^−1^) of 30% In_2_O_3_/BiOCl are slightly reduced. However, it overcomes the drawback of In_2_O_3_, which can only absorb light above 360 nm without any PFOA degradation activity (verified by experimental exploration in subsequent mechanism research studies).

#### 3.1.2 XPS analysis of photocatalysts

**Fig. 5:**
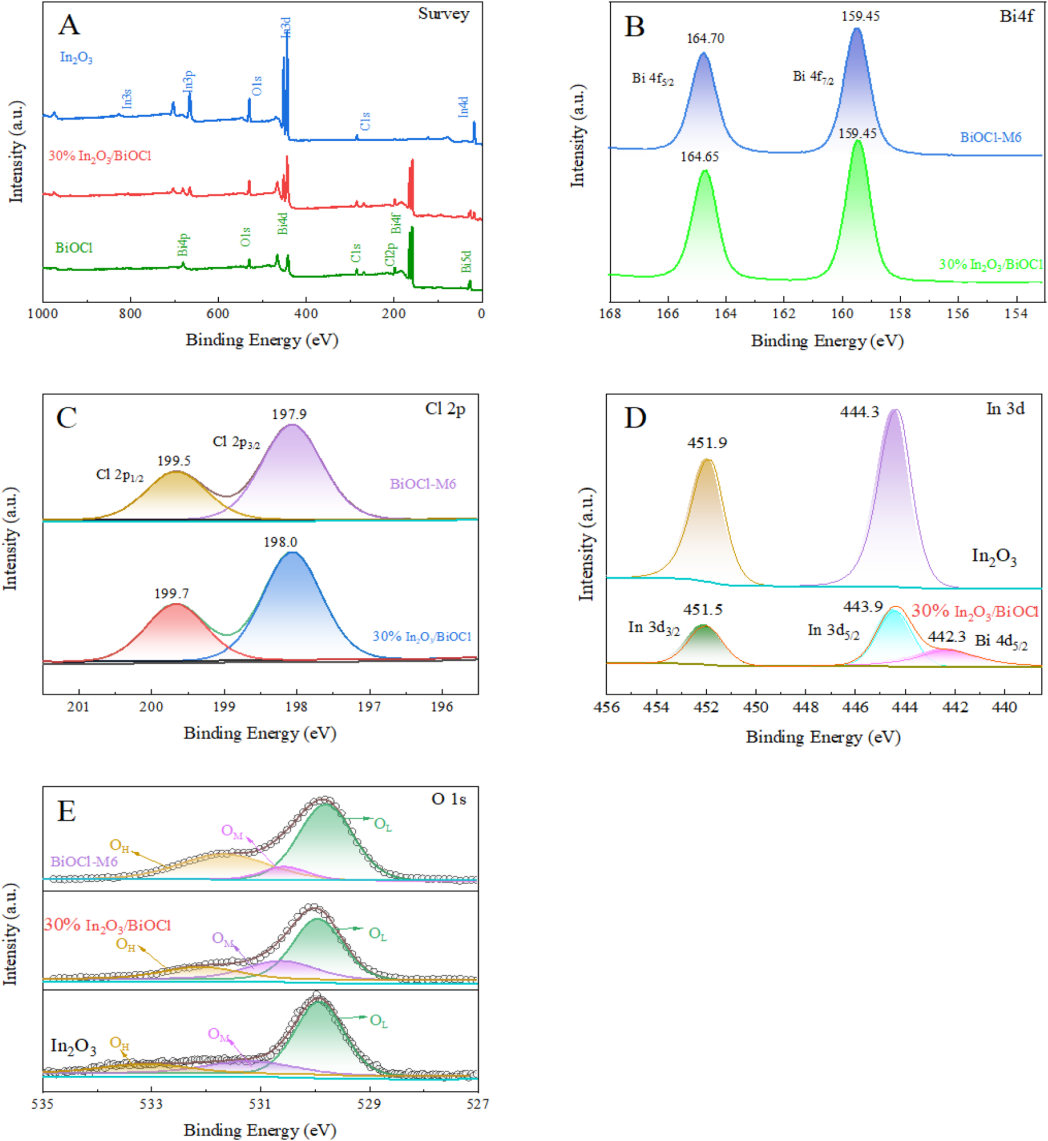
XPS spectra of the photocatalysts. (A) XPS spectra of BiOCl, In_2_O_3_, and 30% In_2_O_3_/BiOCl; (B) Bi 4f spectra of BiOCl and 30% In_2_O_3_/BiOCl; (C) Cl 2p spectra of BiOCl and 30% In_2_O_3_/BiOCl; (D) In 3d spectra of In_2_O_3_ and 30% In_2_O_3_/BiOCl; and (E) O 1s spectra of BiOCl, In_2_O_3_, and 30% In_2_O_3_/BiOCl.

The surface chemical composition and valence state of the heterojunction were extensively studied using X-ray photoelectron spectroscopy (XPS), as shown in Fig. 5. Fig. 5(A) displays the XPS spectra of In_2_O_3_, 30% In_2_O_3_/BiOCl, and BiOCl. The presence of elements In, Bi, Cl, and O is distinctly observed in the XPS spectrum of the 30% In_2_O_3_/BiOCl heterojunction. Fig. 5(B) displays the high-resolution XPS spectrum of Bi 4f. These two dominant peaks at 164.6 eV and 159.4 eV correspond to the Bi 4f_5/2_ and Bi 4f_7/2_ signals of BiOCl, respectively.^37–39^ The two characteristic peaks of Bi 4f on In_2_O_3_/BiOCl shift to 164.7 eV and 159.5 eV, respectively, which may be attributed to the coupling interface interaction between the heterogeneous components. Fig. 5(C) shows the high-resolution XPS spectrum of Cl 2p. The binding energies of the two strong peaks at 198.1 eV and 199.7 eV are assigned to Cl 2p_1/2_ and Cl 2p_3/2_ of Cl in BiOCl, respectively.^40,41^ Compared with pure BiOCl, the binding energies of both characteristic peaks from In_2_O_3_/BiOCl slightly shifted towards higher energy, providing evidence of the coupling interface interaction caused by the formation of the heterojunction. As shown in Fig. 5(D), In 3d of In_2_O_3_ is located at 451.9 eV and 444.3 eV, corresponding to the In 3d_3/2_ and In 3d_5/2_ peaks, respectively.^42,43^ The two In 3d peaks of the In_2_O_3_/BiOCl heterojunction are located at 451.5 eV and 443.9 eV, respectively, which shifts towards lower binding energy compared to pure In_2_O_3_. This indicates that electrons on In_2_O_3_ transfer to BiOCl, forming an interface electric field at the coupling interface. Moreover, a small peak appears at 442.3 eV in Fig. 5(D) for In_2_O_3_/BiOCl, which can be attributed to Bi 4d_5/2_ of BiOCl and further confirms the formation of the heterojunction.^44^ From Fig. 5(E), it can be observed that each O 1s peak can be deconvoluted into three peaks. The peak with the lowest binding energy can be attributed to the Bi–O and In–O chemical bonds, oxygen atoms in the lattice of a molecule (O_L_). The peak with higher binding energy corresponds to an oxygen atom in the hydroxyl group of the adsorbed molecule (O_H_). The peak with the highest binding energy is typically associated with oxygen ions that contain defects in the imperfect lattice (O_M_).^45–47^ It is observed that the O 1s fitting peaks in the heterojunction shifted towards lower binding energies to a certain extent, indicating the intimate interfacial contact between neighboring components. This is consistent with the result from the XRD spectroscopic analysis, which confirms the successful fabrication of the In_2_O_3_/BiOCl heterojunction. Furthermore, as shown in the fitting curves and Table 3, the percentage of the oxygen-vacancy-rich region in the In_2_O_3_/BiOCl heterojunction is higher than that in BiOCl, indicating an abundance of oxygen vacancies. These vacancies can serve as adsorption sites and facilitate the chemical adsorption of oxygen molecules at relatively low temperatures. Furthermore, the appropriate oxygen vacancies can enhance the photocatalytic performance by capturing electrons and suppressing the recombination of photo-generated electron–hole pairs,^48^ thereby improving the utilization of light.

**Table 3:** Sample O 1s peak fitting results

Sample	O_L_ atomic (%)	O_M_ atomic (%)	O_H_ atomic (%)	O_M_/O_L_
In_2_O_3_	61.69	7.21	31.10	0.12
BiOCl	65.12	19.06	15.82	0.29
In_2_O_3_/BiOCl	57.87	25.01	17.12	0.43

#### 3.1.3 Adsorption of PFOA

**Fig. 6:**
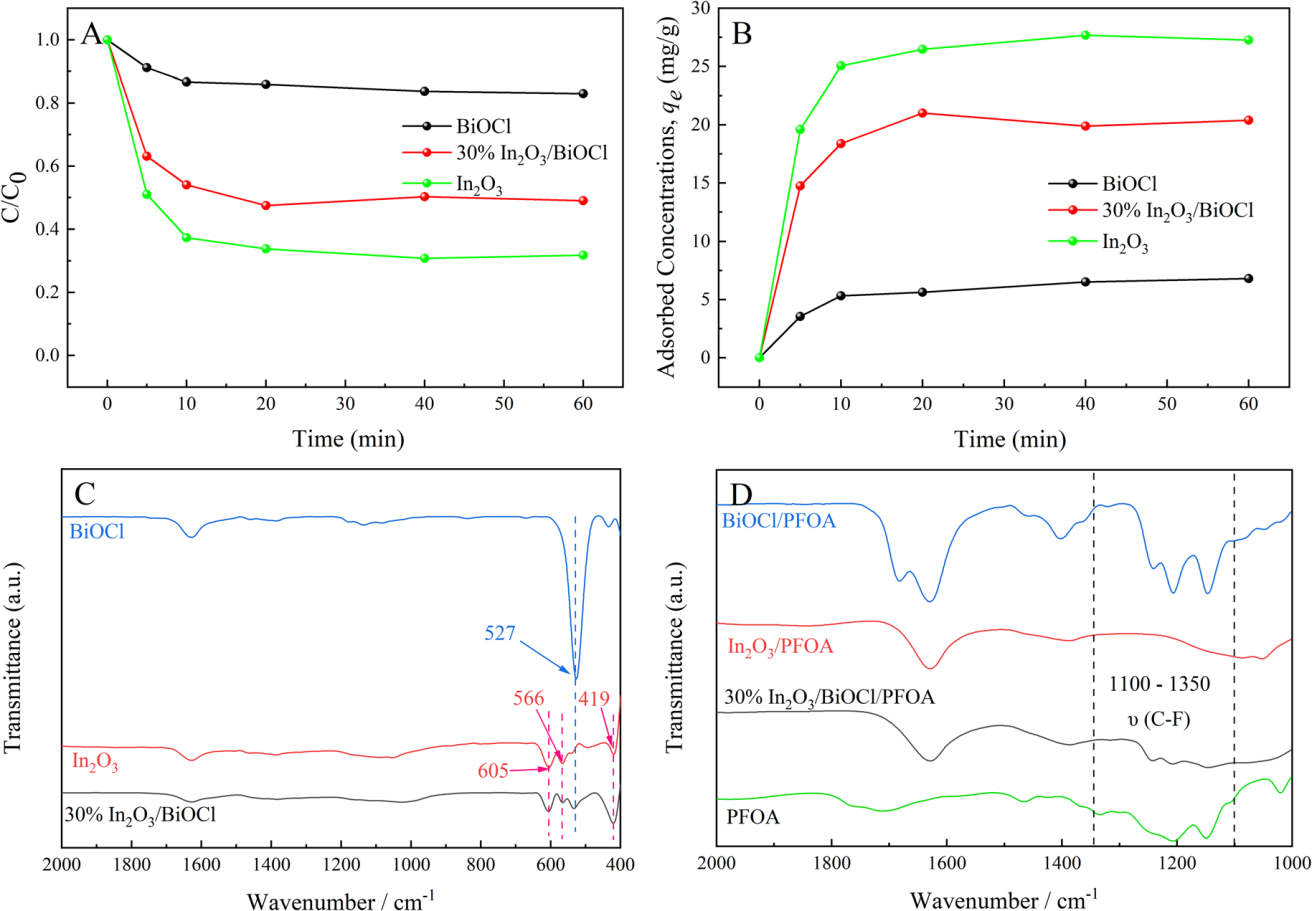
Adsorption curve and FT-IR spectra. (A) Adsorption curve of different photocatalysts. (B) Adsorption volume change curve. (C) FT-IR spectra of BiOCl, In_2_O_3_, and 30% In_2_O_3_/BiOCl. (D) FT-IR spectra of BiOCl/PFOA, In_2_O_3_/PFOA, and 30% In_2_O_3_/BiOCl/PFOA.

The adsorption performance of the photocatalyst on PFOA was investigated because having strong adsorption is beneficial for the photocatalytic reaction.^49^ The adsorption of PFOA on In_2_O_3_, In_2_O_3_/BiOCl, and BiOCl is shown in Fig. 6(A and B). Adsorption experiments were conducted by adding 25 mg of different photocatalysts to 50 mL (20 ppm) of PFOA solution. Fig. 6(A and B) demonstrates that the initial 20 min correspond to the rapid adsorption stage, followed by a decrease in the adsorption rate of PFOA by photocatalysts. The rapid adsorption can be attributed to two factors: (1) the abundance of oxygen vacancies on the BiOCl material surface allows for the adsorption of carboxyl oxygen atoms of PFOA, and the formation of chemical bonds; (2) In_2_O_3_ prepared by the sacrificial MOF framework contains numerous inner channels, which facilitates the adsorption and diffusion of molecules and possession of a high specific surface area, promoting the interaction between the material and reactants.^50^ After 60 min, the maximum adsorption capacities of BiOCl, In_2_O_3_, and 30% In_2_O_3_/BiOCl are 17.0%, 68.2%, and 51.0%, respectively. The corresponding maximum adsorption capacities are determined to be 6.815 mg g^−1^, 27.272 mg g^−1^, and 20.404 mg g^−1^, respectively. To gain a better understanding of the adsorption mechanism, fitting analysis was performed on Fig. 6(B). The rate constants and correlation coefficients for the pseudo-first-order and pseudo-second-order kinetics models were calculated for different photocatalysts based on the experimental data, as shown in Table 4. The high values of the correlation coefficient *R*^2^ in the table indicate that the adsorption of PFOA on the surface of the photocatalyst of BiOCl involves both physical and chemical adsorption. For BiOCl, the correlation coefficients (*R*^2^) obtained from the fitting analysis for pseudo-first-order and pseudo-second-order kinetics are 0.985 and 0.992, respectively. The pseudo-second-order kinetics model shows a higher correlation coefficient. The adsorption process of PFOA on BiOCl is more accurately described by the pseudo-second-order kinetic model, indicating that the adsorption of PFOA on the photocatalyst mainly occurs through chemical adsorption. However, in the case of 30% In_2_O_3_/BiOCl and In_2_O_3_, the correlation coefficient of the pseudo-first-order kinetic model is greater than that of the pseudo-second-order kinetic model, suggesting that the pseudo-first-order kinetic model can more accurately describe the adsorption process of both photocatalysts on PFOA, and physical adsorption is the main mechanism. Moreover, the corresponding kinetic model equations can be used to more accurately calculate the adsorption capacity of photocatalysts for PFOA at equilibrium.^49^

**Table 4:** Adsorption kinetic parameters of PFOA on BiOCl, 30% In_2_O_3_/BiOCl and In_2_O_3_

Adsorbent	Pseudo first-order			Pseudo second-order		
	*q*_e_ (mg g^−1^)	*K*_1_ (min^−1^)	*R* ^2^	*q*_e_ (mg g^−1^)	*K*_2_ (g mg^−1^ min^−1^)	*R* ^2^
BiOCl	6.487	0.157	0.985	7.295	0.029	0.992
30% In_2_O_3_/BiOCl	20.401	0.252	0.997	21.640	0.023	0.987
In_2_O_3_	27.209	0.253	0.999	28.999	0.016	0.994

FT-IR spectroscopy was utilized to further determine the composition of BiOCl, In_2_O_3_, and 30% In_2_O_3_/BiOCl and their adsorption capacity for PFOA, and the results are shown in Fig. 6. Fig. 6(C) reveals that the characteristic peak of pure BiOCl at 527 cm^−1^ is attributed to the stretching vibration of the Bi–O bond.^51^ The characteristic peaks of pure In_2_O_3_ at 605 cm^−1^, 566 cm^−1^, and 419 cm^−1^ can be attributed to the asymmetric stretching of In–O bond.^52–54^ Furthermore, the distinctive peaks of pure BiOCl and In_2_O_3_ can be observed in the FT-IR spectra of the 30% In_2_O_3_/BiOCl heterojunction, indicating the successful preparation of heterojunction, which is consistent with the XRD results. In order to investigate the adsorption of PFOA on the photocatalyst, the infrared spectra of the photocatalyst after PFOA adsorption were analyzed. As shown in Fig. 6(D), the peaks from 1100 cm^−1^ to 1350 cm^−1^ are attributed to the vibrations of the –CF_3_ and –CF_2_ groups of PFOA.^55^ Upon adsorption of PFOA onto BiOCl and 30% In_2_O_3_/BiOCl heterojunction, the corresponding peaks for the –CF_3_ and –CF_2_ groups of PFOA are observed from 1100 cm^−1^ to 1350 cm^−1^. However, after PFOA is adsorbed onto In_2_O_3_, no corresponding vibration peak is found from 1100 cm^−1^ to 1350 cm^−1^. This may be attributed to the removal of the MOF framework during the preparation of In_2_O_3_, which creates a significant number of porous structures that facilitate the internal adsorption of most PFOA molecules in the photocatalyst. Consequently, the concentration of residual PFOA on the surface of In_2_O_3_ is relatively low, making it undetectable by infrared spectroscopy. The reaction rate of the pollutant adsorbed in the internal pores of the photocatalyst is limited by diffusion, leading to a lower reaction rate. Surface adsorption leads to the proximity of the reactant molecules to the active sites on the photocatalyst surface, and establishes an adsorption–desorption equilibrium state. This proximity and equilibrium state can promote the reaction rate. These results provide a foundation for the high efficiency of BiOCl in the degradation of PFOA.

### 3.2 Investigation of the catalytic activity and stability of the heterojunction

**Fig. 7:**
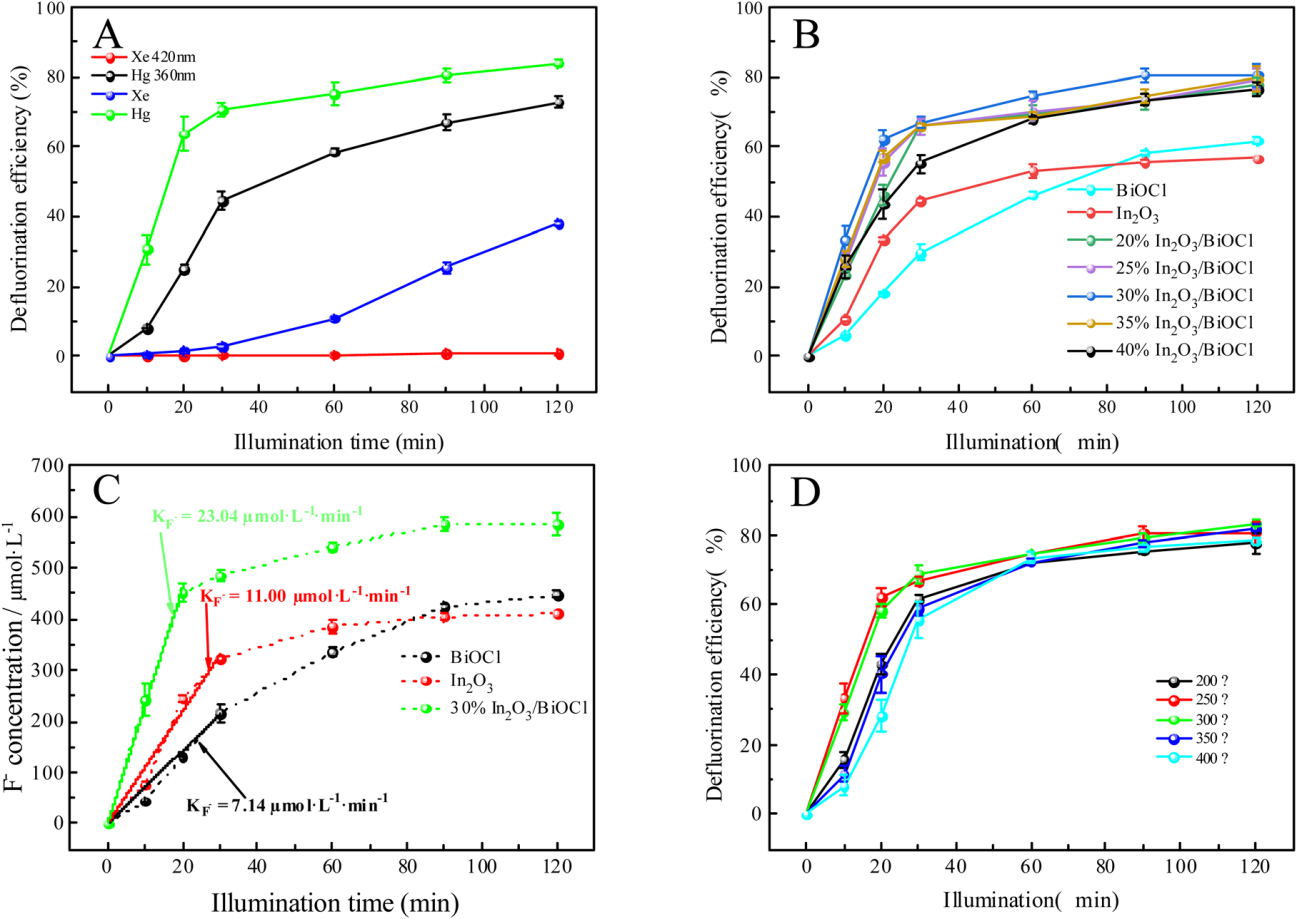
Effect of preparation conditions of the photocatalyst on its photocatalytic activity. (A) The effect of different wavelengths of light source on the catalytic activity. (B) The effect of different composite ratios on the activity of the photocatalyst. (C) Pseudo-first-order defluorination rates of different photocatalysts. (D) The effect of calcination temperature on the catalytic activity.

The photodegradation of PFOA was investigated under the fixed initial PFOA concentration of 20 mg L^−1^, pH of 4, and photocatalyst dosage of 0.1 g L^−1^, as presented in Fig. 7. To obtain the complete PFOA degradation process, the photocatalytic activity of the 30% In_2_O_3_/BiOCl heterojunction was studied under a different light source, as illustrated in Fig. 7(A). Results show that the defluorination efficiency and rate of PFOA both decrease with increasing light source wavelength. At 420 nm wavelength, the PFOA degradation becomes negligible due to the photocatalyst's excessively wide bandgap, which hinders the efficient utilization of visible light. Therefore, a mercury lamp with an emission wavelength of 360 nm was adopted as a light source for subsequent experiments. The photocatalytic activity of BiOCl, In_2_O_3_, and different composite ratios of In_2_O_3_/BiOCl under UV light was investigated. As shown in Fig. 7(B), it can be seen that the defluorination rate of PFOA by BiOCl and In_2_O_3_ is 61.82% and 56.69%, respectively, while the defluorination efficiency of PFOA exceeds 75% by the heterojunction photocatalyst. Especially, the defluorination rate of the 30% In_2_O_3_/BiOCl photocatalyst reaches 80.81%, indicating that the photocatalytic activity is significantly enhanced. The amount of fluoride generated during the degradation increases linearly within 30 min (Fig. 7(C)). The reaction is determined to be the pseudo-zero-order reaction. The slope of the graph calculates the fluoride generation rate of 23.04 μmol L^−1^ min^−1^ in the heterojunction photocatalyst In_2_O_3_/BiOCl system, which is 3.23 times (7.14 μmol L^−1^ min^−1^) and 2.09 times (11.00 μmol L^−1^ min^−1^) higher than the fluoride generation rate of pure BiOCl and the pure In_2_O_3_ photocatalyst, respectively. Compared with the pure BiOCl and pure In_2_O_3_ photocatalysts, the heterojunction system does not possess any special active sites. Moreover, the specific surface area of the heterojunction (13.87 m^2^ g^−1^) is inferior to that of In_2_O_3_ (33.09 m^2^ g^−1^). Nevertheless, the excellent defluorination performance of the In_2_O_3_/BiOCl heterojunction photocatalysts toward PFOA is attributed to the establishment of an internal electric field, which enhances the utilization of light by the photocatalyst. Fig. 7(D) illustrates the photocatalytic activity of the 30% In_2_O_3_/BiOCl heterojunction photocatalyst prepared by different calcination temperatures. It can be observed that the activity significantly increases when the calcination temperature is increased from 200 °C to 300 °C, which may be attributed to the more stable interface coupling between BiOCl and In_2_O_3_ at a higher calcination temperature. However, the activity of the heterojunction somewhat decreases when the calcination temperature further increases to 400 °C, owing to the limited thermal stability of BiOCl. Its activity decreases when the calcination temperature exceeds 300 °C, leading to the decreasing photocatalytic activity of the heterojunction.

**Fig. 8:**
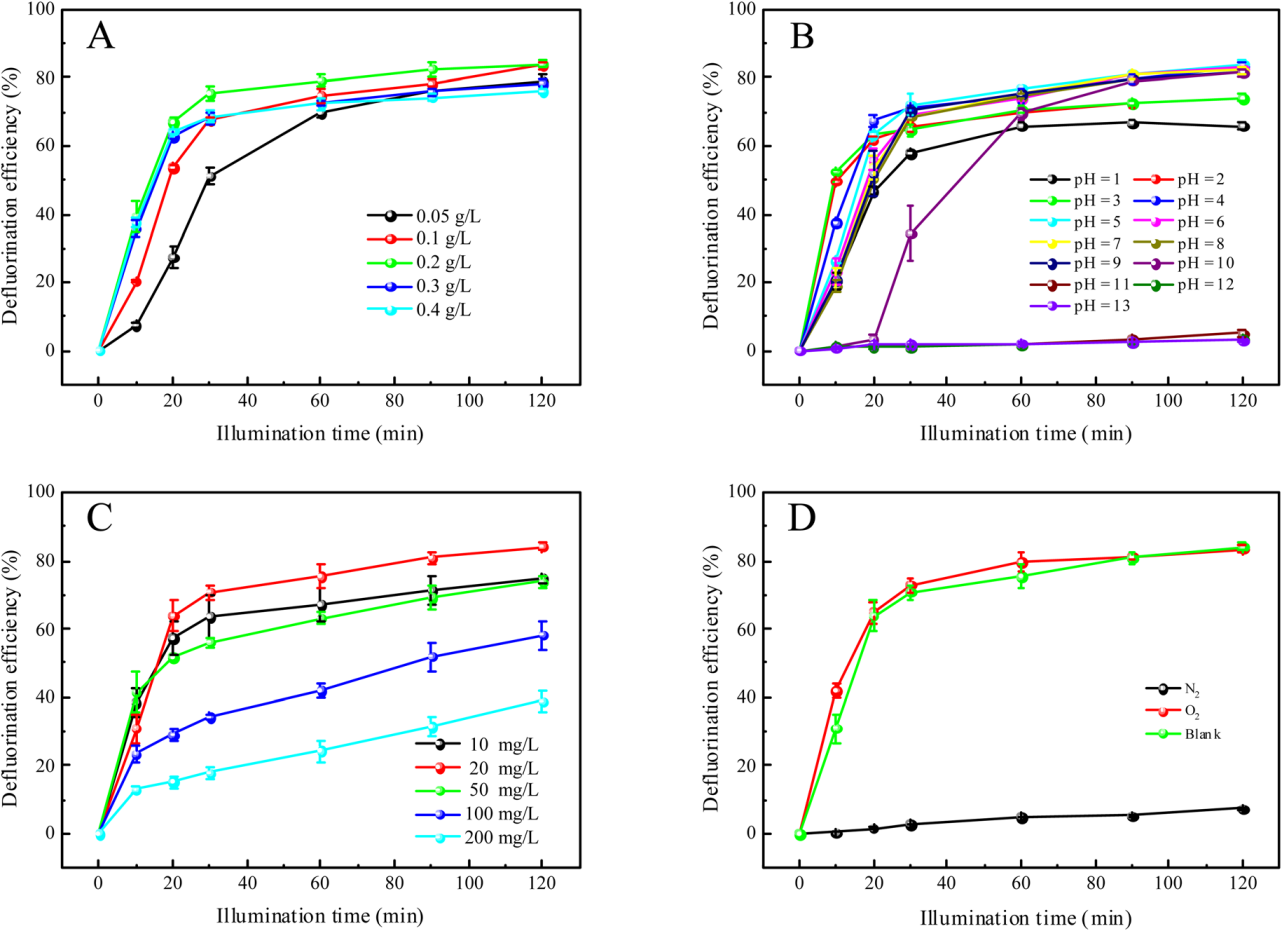
Effect of photocatalytic degradation conditions on the defluorination rate of PFOA. (A) The effect of catalyst loading on the defluorination rate. (B) The effect of degradation solution pH on the defluorination rate. (C) The effect of the concentration of the degradation product on the defluorination rate. (D) The effect of different environmental atmospheres on the defluorination rate.

To investigate the optimal catalytic degradation process of the photocatalyst, PFOA was used as the reaction substrate, and the effect of the photocatalyst dosage, initial pH value of pollutant and concentration of the degradation product on the degradation efficiency of the heterojunction photocatalyst were systematically investigated, as shown in Fig. 8. When the initial concentration of PFOA is 20 mg L^−1^ and pH is 4, different dosages of the 30% In_2_O_3_/BiOCl heterojunction photocatalyst are used to degrade PFOA. As shown in Fig. 8(A), the degradation efficiency continuously increases with increasing catalyst dosage within the range of 0.05 g L^−1^ to 0.2 g L^−1^. This phenomenon can be attributed to the fact that with a few photocatalysts, the availability of the catalytically active sites is limited, whereas with increasing dosage of photocatalyst, the quantity of active sites becomes more abundant.^56^ However, upon increasing the dosage of the photocatalyst, the degradation efficiency of PFOA presents a downward trend. The reason is that the excessive amount of photocatalyst results in photocatalyst aggregation, which increases the solution turbidity, hinders the transmission of radiation to the interior of the solution, and scatters light, thereby reducing the efficiency of the photocatalytic degradation.^57^ Therefore, 0.2 g L^−1^ is chosen as the optimal dosage of the photocatalyst.

The pH of the degradation solution plays a critical role in determining the surface charge properties of the catalyst and affects the adsorption of reactants on the catalyst surface, thereby influencing the efficiency of the photocatalytic reactions.^58^ Therefore, in the process of the photocatalytic degradation of PFOA, the initial concentration of PFOA was fixed at 20 mg L^−1^, the catalyst dosage was 0.2 g L^−1^, and the pH was adjusted from 1 to 13. The defluorination efficiency of PFOA is shown in Fig. 8(B). As the pH of the PFOA solution increases from 1.00 to 13.00, the degradation rate of the In_2_O_3_/BiOCl heterojunction photocatalyst firstly increases and then decreases, while the defluorination rate in the first 20 min decreases gradually with the increase of pH. It suggests that the acidic conditions are conducive to the degradation of PFOA. When the pH value is between 1.0 and 3.0, the defluorination effect is poor. This is attributed to the influence of NO_3_^−^ in the nitric acid solution used to adjust the pH. Furthermore, the evaporation of HF gas caused by the combination of desorbed F^−^ and H^+^ in solution results in a lower measured concentration of fluoride ion. The photocatalytic degradation of PFOA reaches its optimum effect when the pH value is 5.0. Therefore, 5.0 is selected as the pH of the degradation solution.

During photocatalytic degradation, the substrate concentration and environmental atmosphere are important factors that influence the degradation. The impact of different initial concentrations of PFOA solutions on the defluorination rate was investigated when the catalyst dosage was 0.2 g L^−1^ and the pH value was 5, as shown in Fig. 8(C). The defluorination rate increases when the concentration of PFOA is increased from 10 to 50 mg L^−1^. This may be attributed to the slow diffusion of PFOA in the solution under a low initial concentration, which makes it difficult to be adsorbed onto the surface of the catalyst. As a result, the defluorination rate increases when the PFOA concentration increases. When the initial concentration of PFOA is in the range of 50–200 mg L^−1^, the defluorination rate decreases with the increase of the initial concentration. This phenomenon may be attributed to the saturation of active sites on the catalyst surface under the low solution concentration. As the solution concentration increases, the relative reduction of active sites leads to a decrease in the defluorination rate. This study also shows that the defluorination rate decreases when the initial concentration increases from 20 to 200 mg L^−1^. The reason is that some F^−^ generated during the reaction will be adsorbed on the active sites, leading to a decrease in the defluorination rate.^28^ The environmental atmosphere during the photocatalytic degradation also affects the reaction efficiency. As shown in Fig. 8(D), it can be seen that the photocatalytic efficiency is enhanced in the presence of a sufficient amount of oxygen, while PFOA is hardly degraded in the nitrogen atmosphere. This suggests that oxygen plays an indispensable role in the degradation process of PFOA.

**Fig. 9:**
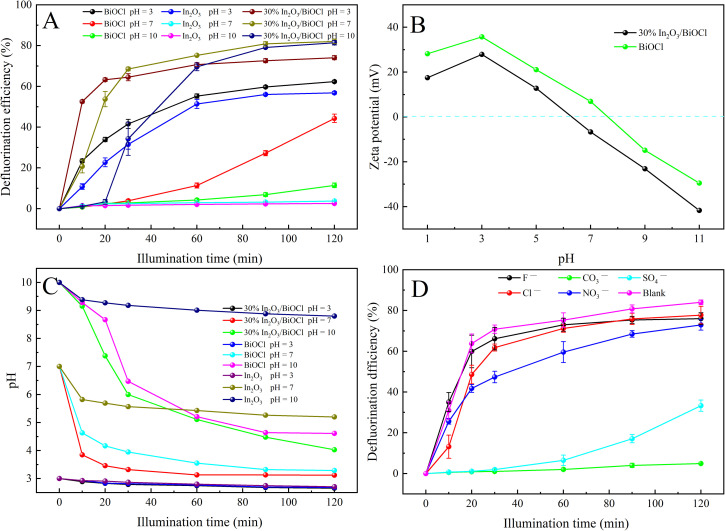
Comparison of PFOA defluorination by different photocatalysts. (A) The influence of various photocatalysts on the defluorination rate under different acidic conditions. (B) Zeta potential at pH 1.00–11.00. (C) pH variation during PFOA degradation with different photocatalysts under different initial pH conditions. (D) The impact of different anions on the defluorination rate.

To demonstrate the superior performance of the heterojunction photocatalyst compared to pure BiOCl and In_2_O_3_, the degradation of PFOA was carried out under acidic (pH = 3.0), neutral (pH = 7.0), and alkaline (pH = 10.0) conditions using different photocatalysts. As shown in Fig. 9(A), the heterojunction exhibits excellent degradation efficiency towards PFOA under acidic (pH = 3.0), neutral (pH = 7.0), and alkaline (pH = 10.0) conditions, while pure BiOCl only shows good degradation efficiency towards PFOA under acidic (pH = 3.0) conditions, with a significant reduction in the degradation activity under neutral (pH = 7.0) conditions, and almost complete suppression of degradation activity under alkaline (pH = 10.0) conditions. For the pure In_2_O_3_ photocatalyst, the pH of the degradation solution has a greater impact on the degradation activity of PFOA, exhibits degradation activity towards PFOA only under acidic conditions (pH = 3.0), and the photocatalytic activity is severely suppressed under neutral (pH = 7.0) and alkaline (pH = 10.0) conditions. To demonstrate the applicability of the heterojunction in a wider pH range, HNO_3_ and NaOH solutions are utilized to adjust the pH of the degradation solution. The zeta potential of pure BiOCl and 30% In_2_O_3_/BiOCl is measured within the pH range of 1.00–11.00. As shown in Fig. 9(B), it can be observed that the zeta potential of pure BiOCl and 30% In_2_O_3_/BiOCl varies at different pH values, but the overall trend in the potential change is consistent between these two materials. However, the zero point of the 30% In_2_O_3_/BiOCl photocatalyst is 6.25. This is lower than the zero point of pure BiOCl (7.60), and inconsistent with the result shown in Fig. 9(A). To explain this phenomenon, the pH is measured during the PFOA degradation process. As shown in Fig. 9(C), it can be observed that the pH of the PFOA solution gradually decreases during the degradation process. Furthermore, the pH of the degradation solution decreases faster in the alkaline environment. This might be because OH^−^ in the degradation solution is oxidized to form ˙OH by hole, and there are more OH^−^ in the alkaline environment, which is more easily oxidized, thus resulting in a faster decrease in pH of the degradation solution. The comparison of different photocatalysts revealed that the pH of the solution in the heterojunction decreases the fastest, while the pH of the pure In_2_O_3_ decreases the slowest. This suggests that the heterojunction photocatalyst exhibits the highest light utilization efficiency and the strongest photocatalytic activity. Based on Fig. 9(A), the degradation of PFOA by the In_2_O_3_/BiOCl heterojunction photocatalyst begins after irradiating for 20 min when the pH value is 10.0. This is because in the first 20 min of irradiation, the pH of the solution is greater than 7.0. Negative charges accumulate on the surface of the catalyst under this condition, making it difficult for the carboxyl groups to be adsorbed with the negative charges at the end of PFOA. When the irradiation time reaches 30 min and the pH value is 6.0, the catalyst surface carries positive charges, allowing them to effectively adsorb PFOA with a negatively charged carboxyl group at the end, resulting in its degradation. However, under the same conditions, BiOCl cannot degrade PFOA until the pH of the degradation solution is lower than 7.0. This may be because the recombination of BiOCl's photogenerated electron–hole pairs is too fast, resulting in a low utilization efficiency of light, so the degradation of PFOA requires a lower pH environment.

To investigate the applicability of In_2_O_3_/BiOCl in real environments, the effect of common anions (1 mM Cl^−^, CO_3_^2−^, NO_3_^−^, SO_4_^2−^, *etc.*) on the photocatalytic degradation of PFOA in water was examined. As shown in Fig. 9(D), all anions exhibit inhibitory effects on PFOA degradation. The weakest inhibitory effect is observed for Cl^−^ and F^−^, while SO_4_^2−^ showed a relatively strong inhibitory ability. The inhibition of PFOA degradation by all three anions can be understood, as these anions occupy part of the active sites and inhibit the degradation process by competing with PFOA for photoinduced holes. The inhibition of the PFOA degradation by NO_3_^−^ is attributed to its ability to capture part of the electrons,^59–61^ while CO_3_^2−^ almost completely inhibits the degradation of PFOA. This may be attributed to CO_3_^2−^ having a carboxyl group and small molecular size, which can almost completely occupy these active sites that the carboxyl end of PFOA can bind on the surface of the catalyst, resulting in ineffective degradation for PFOA. This indirectly indicates that PFOA is connected to the catalyst through the carboxyl end of PFOA.

**Fig. 10:**
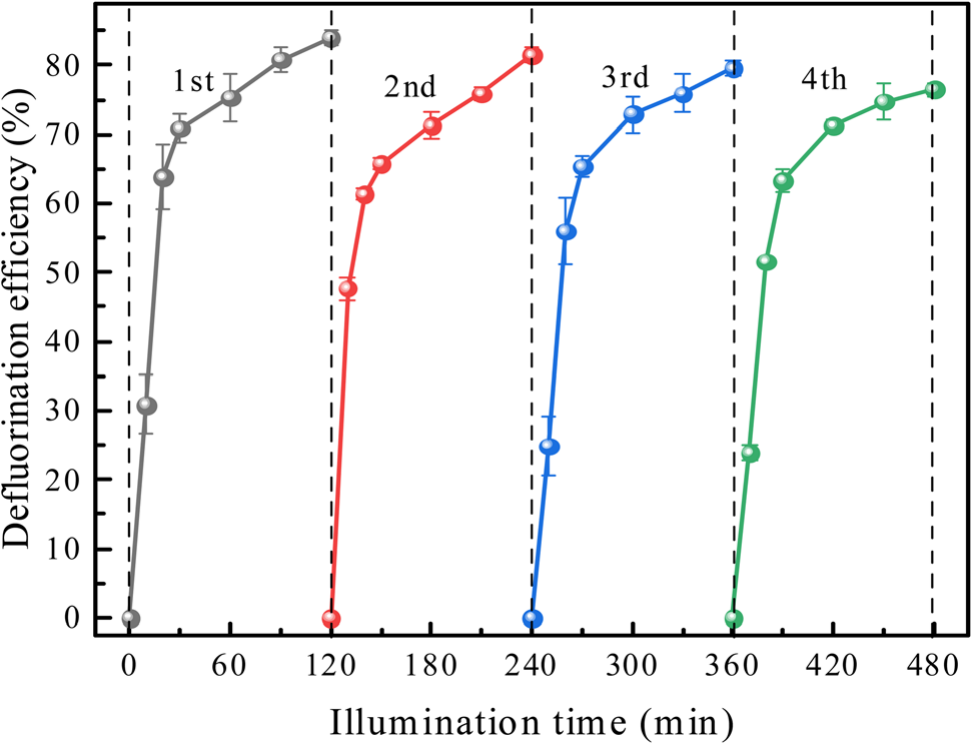
Effect of the reuse of 30% In_2_O_3_/BiOCl on PFOA defluorination.

The stability of 30% In_2_O_3_/BiOCl for the photocatalytic degradation of PFOA was investigated, and the results are shown in Fig. 10. The results showed that even after the catalyst is used four times, the defluorination efficiency of PFOA still reaches 76.44%. Compared with the first use of a catalyst, the defluorination rate of PFO is only reduced by 7.00%, reaching 84.01%. At the same time, the defluorination rate is significantly increased during the first 10 min when 30% In_2_O_3_/BiOCl is reused, which may be related to the increase in the oxygen vacancies in part of BiOCl within the heterojunction under UV-light illumination, leading to a decrease in the recombination rate of the photogenerated electron–hole pairs.

### 3.3 Research on the degradation mechanism of PFOA

#### 3.3.1 Degradation mechanism of PFOA

**Fig. 11:**
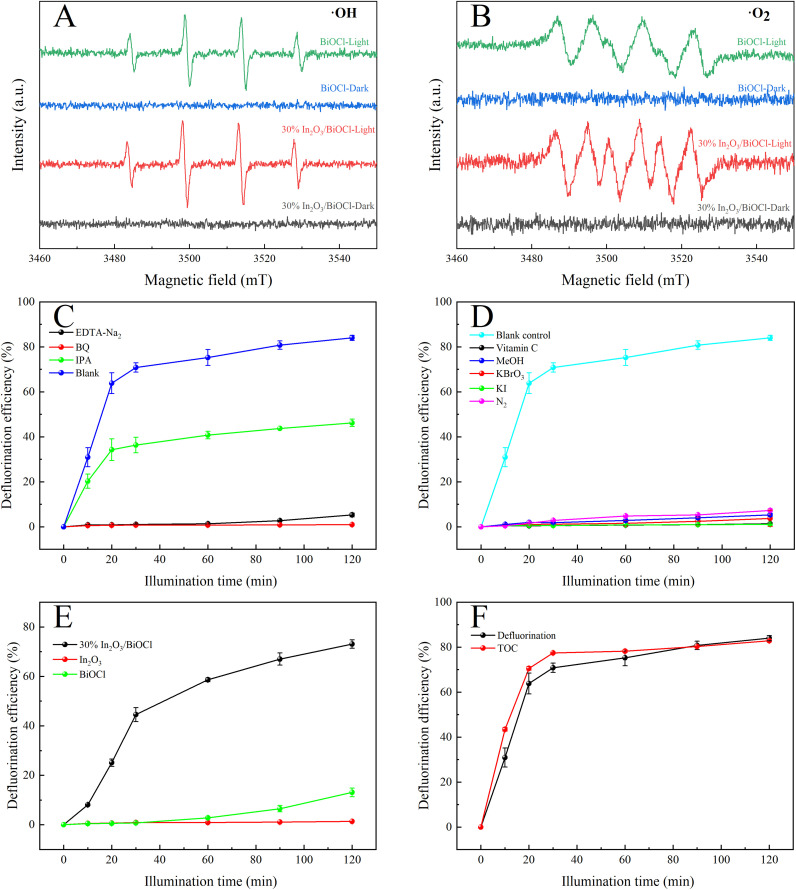
(A and B) ESR spectra of ˙OH and ˙O_2_^−^ of BiOCl and 30% In_2_O_3_/BiOCl. (C) Free radical capture experiment. (D) Control experiment of ˙O_2_^−^ inhibition and h^+^ generation. (E) Degradation efficiency of different photocatalysts on PFOA under a 360 nm filter. (F) TOC removal rate of PFOA.

To investigate the photodegradation mechanism of PFOA by the 30% In_2_O_3_/BiOCl heterojunction, the active species were studied using electron spin resonance (ESR) with 5,5-dimethyl-1-pyrroline-*N*-oxide (DMPO) as a spin trap agent. As shown in Fig. 11(A) and (B), there is almost no ESR signal observed for BiOCl and 30% In_2_O_3_/BiOCl under dark conditions. However, the four-line characteristic ESR signal of the DMPO–˙OH and DMPO–˙O_2_^−^ spin adducts is observed under simulated solar light irradiation, indicating that both catalytic systems can generate ˙OH and ˙O_2_^−^. Furthermore, all ESR signals produced by the 30% In_2_O_3_/BiOCl heterojunction are significantly stronger than those produced by BiOCl, which indicates that more active species can be generated in a 30% In_2_O_3_/BiOCl heterojunction photocatalytic system. To explore the main active substances in the degradation process of PFOA, various scavengers are added to the degradation reaction to study their roles in photocatalysis. Among them, 1 mM EDTA-Na_2_ is used as a hole (h^+^) scavenger, 1 mM IPA is used as a hydroxyl radical (˙OH) scavenger, and 1 mM BQ is used as a scavenger of superoxide radicals (˙O_2_^−^). From Fig. 11(C), it can be seen that when EDTA-Na_2_ and BQ are added as scavengers, the defluorination rate of PFOA decreases significantly under the same conditions from 84.01% without any scavenger to 5.23% and 0.95%, respectively, indicating a significant reduction in the defluorination rate and nearly complete inhibition of the PFOA degradation. This suggests that h^+^ and ˙O_2_^−^ play a major role in the photocatalytic degradation of PFOA. When IPA is introduced as a capture agent under identical experimental conditions, the defluorination efficiency only decreases to 46.23%, indicating that ˙OH only plays a supporting role in the PFOA defluorination process. However, in a photocatalytic system, even though ˙O_2_^−^ can be produced through an electronic reduction pathway, the oxidation ability of ˙O_2_^−^ is too weak to decompose the PFOA molecules.^45^ Therefore, it can be inferred that the defluorination of PFOA occurs under the joint action of h^+^ and ˙O_2_^−^.

Given the influence of substances (such as quinones) on the color of the solution, their presence may obstruct light and thus impact the degradation of PFOA. To investigate this phenomenon, ˙O_2_^−^ scavenger (1 mM ascorbic acid) is selected and utilized to eliminate ˙O_2_^−^, as shown in Fig. 11(D). In addition, by adding 2.5 mM KBrO_3_ to capture electrons and indirectly suppress the formation of ˙O_2_^−^, the control experiments are conducted under an anaerobic N_2_ environment. The results demonstrate significant inhibition of PFOA degradation in all scenarios. The impact of the hollow structure on PFOA degradation is further confirmed through the addition of 1 mM KI and 1 mM MeOH. Fig. 11(E) shows the degradation efficiency of three different materials towards PFOA under UV light above 360 nm. Compared with Fig. 7(B), it can be observed that under irradiation with light at wavelengths above 360 nm, the 30% In_2_O_3_/BiOCl heterojunction still achieves a defluorination efficiency exceeding 70%, while the degradation efficiency of BiOCl is significantly inhibited. In particular, the degradation efficiency of In_2_O_3_ is completely inhibited. This indicates that under light irradiation above 360 nm, the holes generated by In_2_O_3_ cannot degrade PFOA. Therefore, in the heterojunction, the degradation of PFOA occurs on the surface of the BiOCl component, and the holes generated by In_2_O_3_ will transfer to the BiOCl component. The increase in the degradation efficiency is due to the improvement of the separation efficiency of photo-generated electron–hole pairs, effectively preventing their recombination, and is consistent with the FT-IR, EIS, and PL results.

The removal efficiency of the total organic carbon (TOC) of the PFOA solution was analyzed, and the mineralization rate was also calculated, which is of great importance for avoiding secondary pollution in wastewater treatment. As shown in Fig. 11(F), the removal efficiency of TOC increased with the increase in the irradiation time and reached 82.87%. At the same time, after ultraviolet irradiation for 2 h, the defluorination rate reached 84.01%, which is consistent with the removal efficiency of TOC.

#### 3.3.2 The separation efficiency of photo-generated electron–hole pairs

##### 3.3.2.1 UV-vis DRS and VB-XPS of the heterojunction

**Fig. 12:**
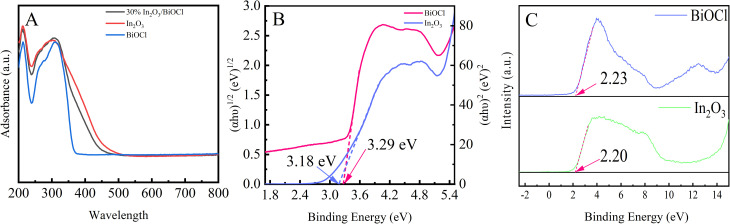
(A) UV-vis DRS spectra of (BiOCl, In_2_O_3_, and 30% In_2_O_3_/BiOCl); (B) (*αhv*)^*n*/2^*vs.* photon energy (*hv*) diagrams of (BiOCl, In_2_O_3_); (C) VB-XPS diagrams of (BiOCl, In_2_O_3_).

UV-vis DRS was employed to reveal the optical properties of BiOCl. To compare the light absorption characteristics of BiOCl, In_2_O_3_, and the 30% In_2_O_3_/BiOCl heterojunction, they were measured by UV-vis DRS (Fig. 12(A)). In_2_O_3_ shows good absorption performance in the UV and visible light regions, and its absorption edge is 500 nm. Pure BiOCl only shows good light absorption ability in the UV region, with an absorption edge at 360 nm. The In_2_O_3_/BiOCl heterojunction demonstrated absorption in the visible light region as well, with an absorption edge at 470 nm. Compared to pure BiOCl, the In_2_O_3_/BiOCl heterojunction exhibits a significantly red-shifted absorption wavelength, which indicates that a broader light absorption range can increase the absorption intensity. The Tauc plot of BiOCl and In_2_O_3_ is calculated and plotted based on the UV-vis DRS data, and the relationship curve between (*αhυ*)^*n*/2^ and *hυ* is shown in Fig. 12(B). The bandgap width (*E*_g_) of the semiconductor can be determined according to its bandgap derivation formula:

1 (*αhυ*)^*n*/2^ = *A*(*hυ* − *E*_g_)

where *α*, *h*, *ν*, *A*, and *E*_g_ are the absorption coefficient, Planck's constant, optical frequency, constant, and bandgap energy, respectively. For the bandgap derivation formula, *n* = 1 in indirect bandgap semiconductors, while *n* = 4 in direct bandgap semiconductors. This graph shows that the bandgap widths of BiOCl and In_2_O_3_ are 3.29 eV and 3.18 eV, respectively. X-ray photoelectron spectroscopy (XPS) can provide information on the electronic structure of the solid material because it is related to the energy band structure of a solid. The valence bands (VB) of BiOCl and In_2_O_3_ photocatalysts are determined by VB-XPS, as shown in Fig. 12(C). The VB edges of BiOCl and In_2_O_3_ are confirmed to be 2.23 and 2.20 eV, respectively. The conduction band position can be estimated according to eqn (2):

2 *E*_CB_ = *E*_VB_ − *E*_g_

The respective conduction band minima of BiOCl and In_2_O_3_ are found to be approximately −1.06 and −0.98 eV.

**Fig. 13:**
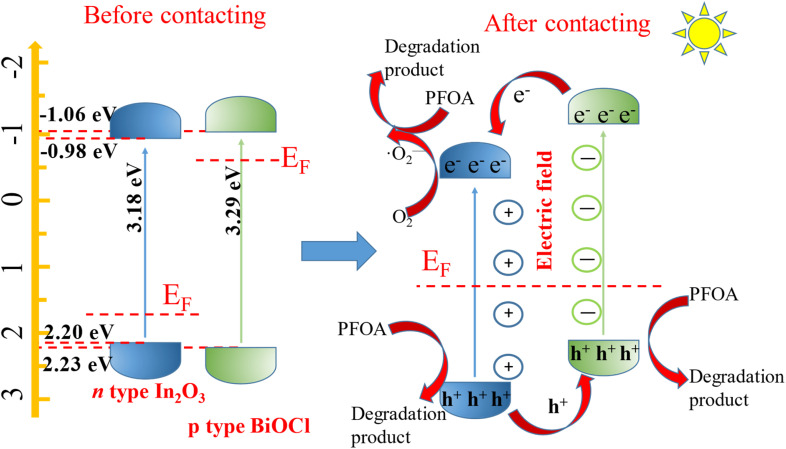
Banded structure of the In_2_O_3_/BiOCl heterojunction.

It is widely believed that p–n heterojunctions can accelerate the separation of photogenerated electron–hole pairs. In the BiOCl@In_2_O_3_ p–n heterojunction, the flow and transfer of electrons can occur at the coupled interface of two semiconductors (Fig. 13). The Fermi energy levels of BiOCl (p-type) and In_2_O_3_ (n-type) are located near VB and CB, respectively. When the interface between the semiconductor materials contacts, the Fermi energy levels of BiOCl (p-type) and In_2_O_3_ (n-type) move upward and downward, respectively, until the Fermi energy level reaches equilibrium, thus establishing an internal electric field. When the BiOCl@In_2_O_3_ p–n heterojunction is exposed to light, photogenerated electrons on the BiOCl conduction band will be transferred to the In_2_O_3_ conduction band, while holes will remain on the VB of BiOCl, thereby effectively separating the photogenerated electron–hole pairs.

##### 3.3.2.2 Electrochemical properties and PL spectra of the heterojunction

**Fig. 14:**
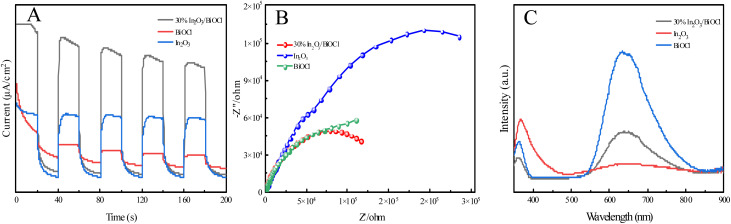
(A) *I*–*t* spectra of (BiOCl, In_2_O_3_, and 30% In_2_O_3_/BiOCl); (B) EIS diagrams of (BiOCl, In_2_O_3_, 30% In_2_O_3_/BiOCl); (C) PL spectra of (BiOCl, In_2_O_3_, 30% In_2_O_3_/BiOCl).

To verify the aforementioned assumption, the transient photocurrent intensity (*I*–*t*), electrochemical impedance spectroscopy (EIS), and photoluminescence spectroscopy (PL) of photocatalysts under simulated visible light irradiation were measured. Fig. 14(A) reflects the photocurrent intensity of these three materials. Generally speaking, the higher the photocurrent response intensity, the higher the separation efficiency of the photo-generated electron–hole pairs.^62^ The photogenerated current intensity of 30% In_2_O_3_/BiOCl is the highest, as indicated in the graph, demonstrating that the formation of the heterojunction effectively enhances the separation efficiency of the photo-generated electron–hole pairs. Conversely, the pure BiOCl material exhibits the lowest photogenerated current intensity, which may be attributed to its wide bandgap that cannot be excited by visible light. This is consistent with the UV-vis diffuse reflectance spectral results. Additionally, all samples have similar trends in the EIS signals. Generally speaking, the smaller the arc radius of the EIS Nyquist plot, the higher the separation efficiency of the photogenerated charge carriers, and the higher the photocatalytic activity.^63,64^ As shown in Fig. 14(B), the EIS Nyquist plot of the 30% In_2_O_3_/BiOCl heterojunction photocatalyst exhibits the smallest arc radius, indicating that 30% In_2_O_3_/BiOCl possesses the highest efficiency for the separation of photogenerated electron–hole pairs. However, in Fig. 14(A) and (B), the test results of In_2_O_3_ and BiOCl show the opposite trend. This discrepancy may be attributed to the larger specific surface area of In_2_O_3_, which can generate more photogenerated carriers and consequently result in a higher transient photocurrent response. Additionally, the larger specific surface area might increase the path length of charge transfer, leading to higher charge transfer resistance. As displayed in Fig. 14(C), the fluorescence spectra of BiOCl, In_2_O_3_ (n-type), and 30% In_2_O_3_/BiOCl were analyzed under the excitation wavelength of 350 nm. All three kinds of materials show emission intensity peaks in the wavelength range between 350–400 nm, which can be attributed to a near-band edge (NBE) UV emission peak resulting from excitonic recombination. Among these materials, the fluorescence intensity of the 30% In_2_O_3_/BiOCl heterojunction is the lowest, suggesting that the rate of the photogenerated electron–hole pair recombination is the slowest for 30% In_2_O_3_/BiOCl. This result is consistent with the results of *I*–*t* and EIS, which demonstrate that the construction of the 30% In_2_O_3_/BiOCl heterojunction effectively inhibits the recombination of the photogenerated electron–hole pairs. The peak located near 550–750 nm may be the deep-level (DL) visible luminescence peak related to structural defects and impurities.^65–67^ It can be observed that the fluorescence intensity of the 30% In_2_O_3_/BiOCl heterojunction is relatively weak compared to that of BiOCl, which also confirms that the effective construction of the 30% In_2_O_3_/BiOCl heterojunction suppresses the recombination of the photo-generated electron–hole pairs.

#### 3.3.3 Detection of intermediate products in PFOA and analysis of the degradation pathways

**Fig. 15:**
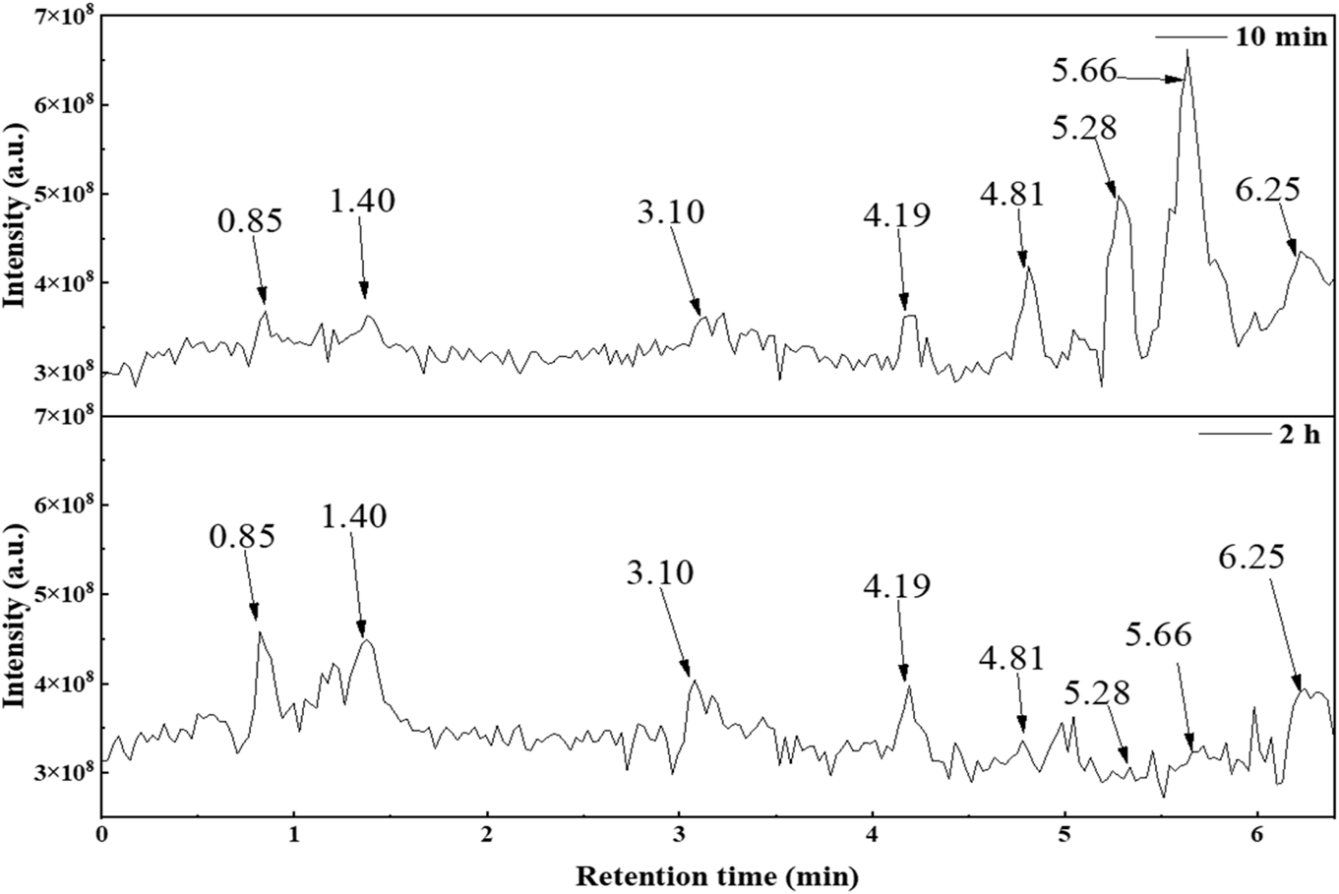
Total ion flow diagram (TIC) of the degradation products of PFOA for different degradation times.

High-performance liquid chromatography/quadrupole time-of-flight mass spectrometry (HPLC/QTOF/MS) was employed to analyze and detect the intermediate products of PFOA degradation. The initial concentration of PFOA was controlled at 20 mg L^−1^, with a degradation solution pH of 4.00 and catalyst dosage of 0.2 g L^−1^. Sampling was conducted for 10 min and 2 h, respectively. The peak-to-peak voltage of 22 kV was utilized to identify the intermediate products using HPLC-QTOF/MS, with molecular weights ranging from 100 to 450. The total ion chromatogram (TIC) is shown in Fig. 15. By selecting the intermediate product peaks with retention times of 0.85, 1.40, 3.10, 4.19, 4.81, 5.28, 5.66, and 6.25 min (other retained peaks may be impurities), it can be seen from the total ion chromatogram of the intermediate products that with the continuous increase in irradiation time from 10 min to 2 h, the intensity and peak area of the intermediate product peaks with elution times of 0.85, 1.40, and 3.10 min have significantly increased, while the intermediate product peaks with elution times of 4.81, 5.28, and 5.66 min have significantly decreased and nearly disappeared. This indicates that molecular ions with elution times of 4.81, 5.28, and 5.66 min are almost completely degraded during the degradation process.

Analysis of the elution peaks on the total ion chromatogram was conducted. The mass spectrometry results are shown in Table 5, where the main chemical substances are 7 kinds of perfluorocarboxylic acids in A_1_, the degradation intermediates in A_2_ and A_3_, and the substances with *m*/*z* ratios of 141, 361, 217, and 285 in A_4_ to A_7_. The other *m*/*z* ratios in A_4_ to A_7_ correspond to substances with the same main characteristic peaks, but differ by adding or reducing one CF_2_ unit. A_8_ and A_9_ show low peak intensity in the mass spectrometry of each elution peak. However, evidence of possible substances can be found in multiple peaks, and their low intensity can be attributed to low molecular concentration. The mass peaks with *m*/*z* ratios of 113, 163, 213, 263, 313, 363, and 413 in the mass spectrum correspond to elution times of 0.85, 1.40, 3.10, 4.19, 4.81, 5.28, and 5.66 min, respectively. Combined with Fig. 15, it can be seen that after an irradiation time of 2 h, elution peaks with elution times of 4.81, 5.28, and 5.66 min almost disappear, indicating that PFOA in the solution is almost completely degraded. Both perfluoroheptanoic acid (PFHpA) and perfluorohexanoic acid (PFHxA), which are degradation intermediates, also disappear, indicating that the heterojunction material 30% In_2_O_3_/BiOCl exhibits excellent performance during the degradation and defluorination of PFOA.

**Table 5:** Determination of intermediates formed at different reaction times using LC/MS

Possible substances	*m*/*z* ([M + H]^−^)	Molecular formula	Molecular structural formula
A_1_	413, 363, 313, 263, 213, 163, 113	C_*n*_F_2*n*+1_COOH	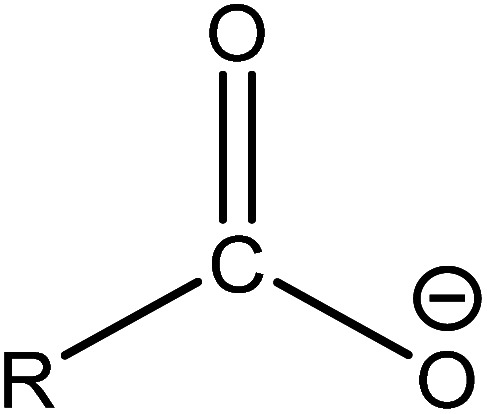
A_2_	369, 319, 269, 219, 169^a^, 119	C_*n*_F_2*n*+1_	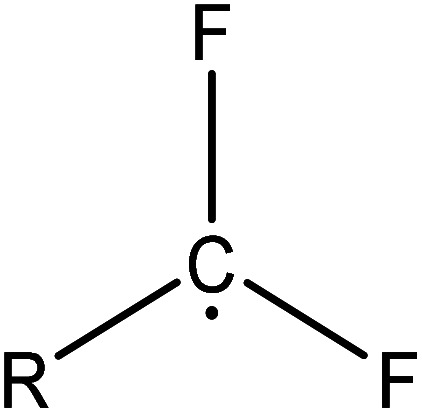
A_3_	347^a^, 297^a^, 247^a^, 197^a^	C_*n*_F_2*n*+1_CO	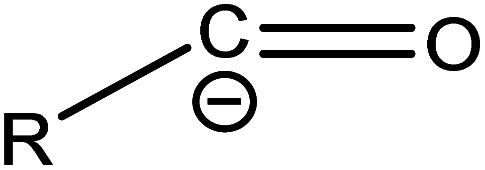
A_4_	391, 341, 141^a^	C_*n*_F_2*n*+1_C_2_O_3_	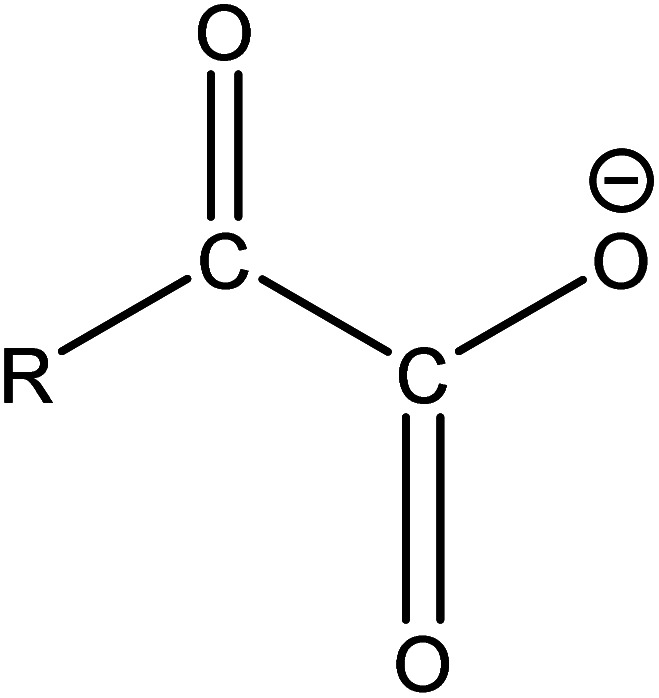
A_5_	361, 311	C_*n*_F_2*n*+1_C_2_FO_3_H	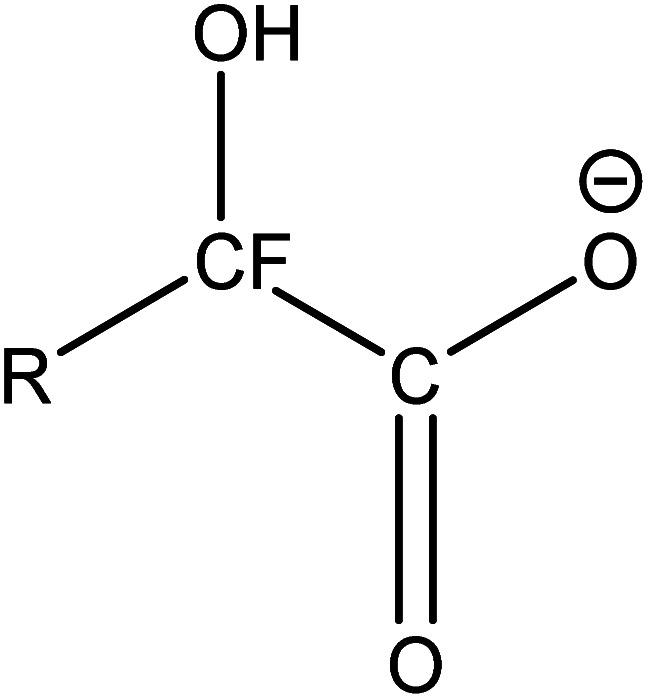
A_6_	367, 217^a^	C_*n*_F_2*n*+1_CFOH	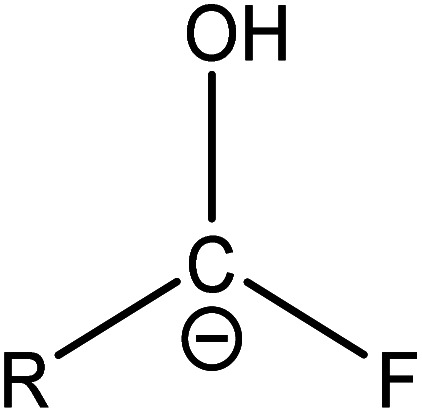
A_7_	335, 285, 235, 185	C_*n*_F_2*n*+1_O	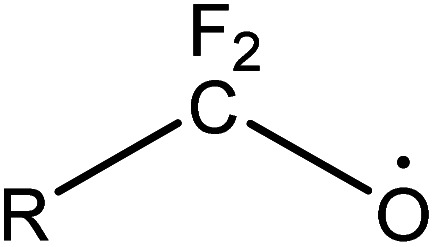
A_8_	401, 301, 201, 151	C_*n*_F_2*n*+1_O_2_	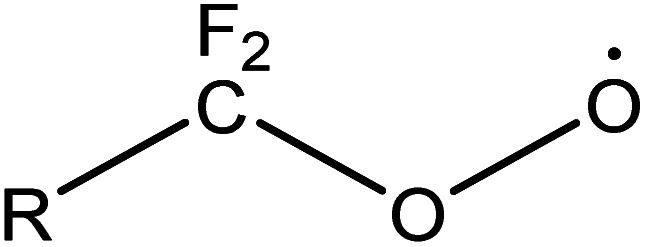
A_9_	409, 359, 309, 259, 209, 109	C_*n*_F_2*n*+1_C_2_O_4_H_2_	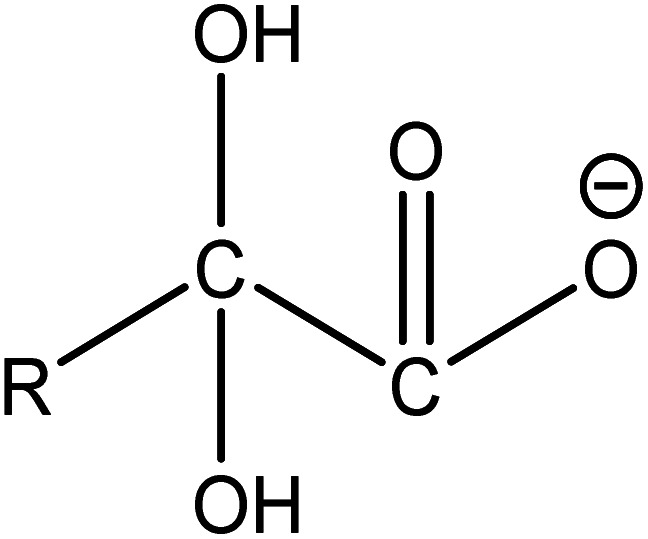

a Peaks of the base ion in each elution time are indicated.

Based on the experimental and characterization analysis results, three kinds of degradation pathways of PFOA are proposed:

(1) The main intermediate product is 
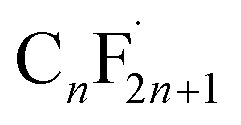
.

Route 1: due to the experimental evidence of free radical capture, ˙OH only plays an auxiliary role during the degradation of PFOA, so there should be only one path for generating 
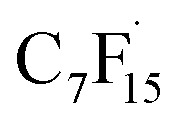
, the reason is that the electrons on the carboxyl end of PFOA adsorbed on the surface of the photocatalyst are captured by h^+^ on the heterogeneous junction surface under light irradiation conditions, generating C_7_F_15_COO˙ (eqn (3)), and then the Photo-Kolbe reaction occurs to remove the carboxyl groups and produce the free radical 
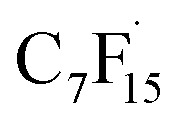
 (eqn (4)).^68^

3 C_7_F_15_COO^−^ + h^+^ → C_7_F_15_COO˙

4
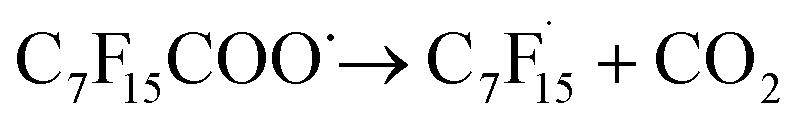


In the further degradation of 
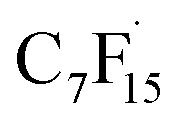
, three reaction pathways have been proposed by scholars. This includes hydrolysis with H_2_O, resulting in the production of C_7_F_15_OH, attacked by ˙OH, resulting in the formation of C_7_F_15_OH, and reaction with O_2_ to generate 
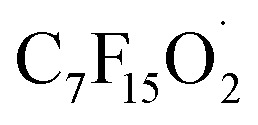
. However, considering previous research that PFOA cannot be defluorinated in a nitrogen atmosphere, these two pathways for 
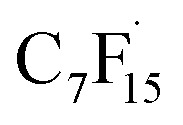
 defluorination through hydrolysis by water and attack by ˙OH are excluded. Therefore, 
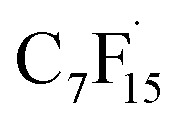
 can only react with O_2_, resulting in the production of the perfluoroperoxyl radical C_7_F_15_OO˙ (eqn (5)), and the subsequent combination of two C_7_F_15_OO˙ molecules to generate oxygen and two kinds of perfluoroalkoxy radicals C_7_F_15_O˙ and O_2_ (eqn (6)).^69^ However, the generated perfluoroalkoxy free radicals can easily react with hydrogen peroxide radicals 
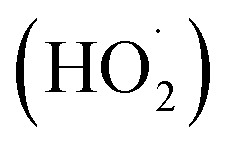
 to form unstable alcohols (eqn (7)), which can easily lose one HF moiety to form C_6_F_13_COF (eqn (8)). C_6_F_13_COF is unstable and easily hydrolyzes to form HF and C_6_F_13_COOH (eqn (9)).5
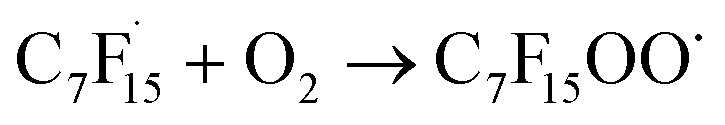

6 C_7_F_15_OO˙ + C_7_F_15_OO˙ → 2C_7_F_15_O˙ + O_2_

7
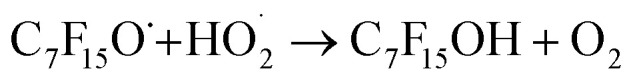

8 C_7_F_15_OH → HF + C_6_F_13_COF

9 C_6_F_13_COF + H_2_O → C_6_F_13_COOH + HF

(2) The main intermediate product is C_*n*_F_2*n*+1_CO.

Route 2: in mass spectrometry analysis, several substances represented by A_3_ are peaks with the highest relative abundance, indicating that they are probably the main intermediates during the degradation of PFOA. As shown in Table 5, the terminal functional group of substances represented by A_3_ is C=O. In order to confirm the origin of the C=O functional group, ion searching was conducted on the total ion chromatograms (TIC) of samples for 10 min and 2 h. It was found that no ion peak with a ratio of mass to charge 397 is detected, which indicates that the C=O functional group in intermediate A_3_ during degradation is not formed by direct dehydroxylation of the carboxyl group at the end of perfluorocarboxylic acid, but by α-C defluorination.

Evidence of free radical capture experiments suggests that both ˙O_2_^−^ and h^+^ play a major role during the degradation of PFOA, in addition to its oxidation capacity. ˙O_2_^−^ is a kind of weakly reducing substance, nucleophilic free radicals that can easily attack low electron density areas in molecules.^70^ Therefore, holes on the surface of the photocatalyst can be seen as electron-withdrawing groups. Under the action of holes, the electron cloud on the alpha carbon moves towards the carboxyl groups, while the electron density on the alpha carbon decreases, making it susceptible to being attacked by nucleophilic free radicals such as ˙O_2_^−^. This leads to a nucleophilic substitution (S_N_2) reaction.^71,72^ The specific process involves the pairing of electrons on ˙O_2_^−^ with electrons on C_7_F_15_CO_2_^−^, followed by electron transference on C_7_F_14_CO_2_^−^ to F atoms attached, resulting in the cleavage of the C–F bond. Additionally, H^+^ provided by the solution leads to intermediates converting into C_6_F_13_CF(HO_2_)COO^−^ (eqn (10)).^72^ Then, hydrogen peroxide decomposes, and the solution provides another H^+^ to generate ˙OH and C_7_F_14_(OH)CO_2_^−^ (eqn (11)). This is one of the reasons why PFOA is more easily degraded under acidic conditions. Then, the end of the carboxyl group is removed to form the unstable C_6_F_13_CHF(OH) (eqn (12)), which easily loses one HF to generate C_6_F_13_CHO (eqn (13)). Finally, it is oxidized by ˙OH and ˙O_2_^−^ to form the corresponding carboxylic acid, C_6_F_13_COOH (eqn (14)).

10 C_7_F_15_COO^−^ + ˙HO_2_^−^ + h^+^ → C_6_F_13_CF(HO_2_)COO^−^ + F^−^

11 C_6_F_13_CF(HO_2_)COO^−^ + H^+^ → C_7_F_14_(OH)CO_2_^−^ + ˙OH

12 C_7_F_14_(OH)CO_2_^−^ + H^+^ + h^+^ → C_6_F_13_CHF(OH) + CO_2_

13 C_6_F_13_CHF(OH) → C_6_F_13_CHO + HF

14 C_6_F_13_CHO + ˙OH/˙O_2_^−^ → C_6_F_13_COOH

Route 3: the generated C_6_F_13_CFO_2_HCOO^−^ directly eliminates HF to form perfluorooctanoic acid (C_6_F_13_COCOOH, eqn (15)),^73^ which further loses the carboxyl group at the end to produce perfluorooctanal (C_6_F_13_CHO, eqn (16)). Eventually, it is oxidized by ˙OH and ˙O_2_^−^ to form the corresponding carboxylic acids (C_6_F_13_COOH, eqn (17)).

15 C_7_F_14_(OH)CO_2_− → C_6_F_13_COCOO^−^ + HF

16 C_6_F_13_COCOO^−^ + H^+^ + h^+^ → C_6_F_13_CHO + CO_2_

17 C_6_F_13_CHO + ˙OH/˙O_2_^−^ → C_6_F_13_COOH

**Fig. 16:**
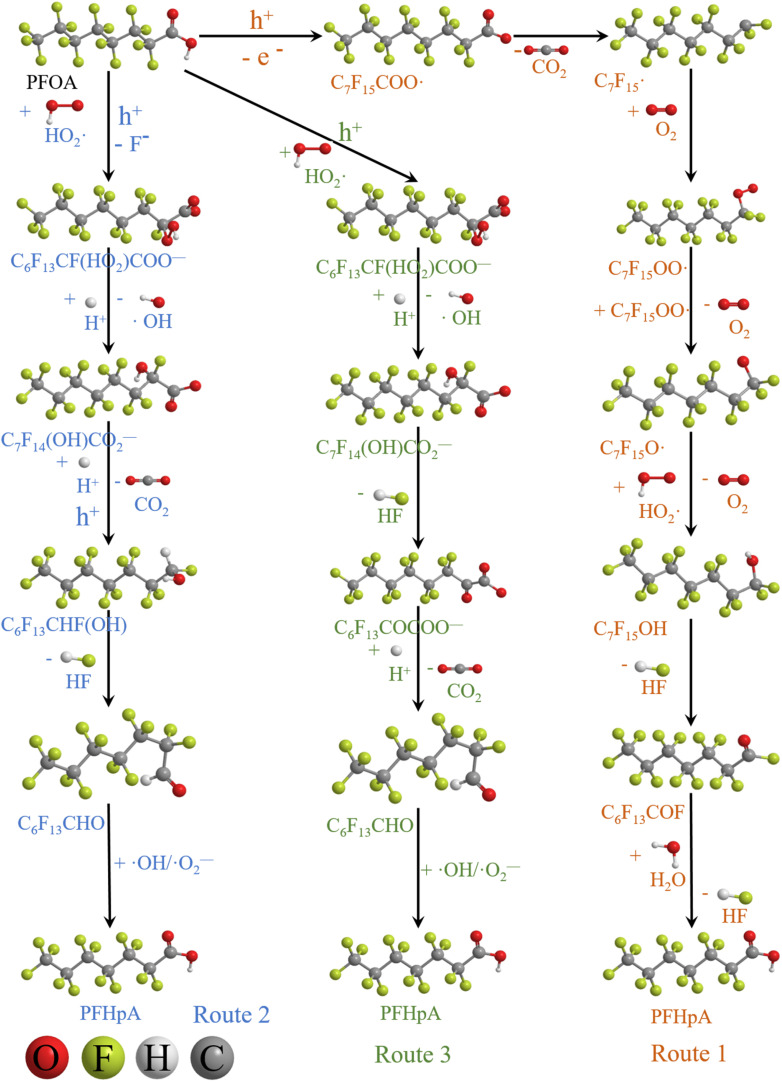
Possible paths of PFOA degradation.

During the photocatalytic degradation of PFOA by the In_2_O_3_/BiOCl heterojunction, the defluorination process is mainly driven by ˙O_2_^−^ and h^+^, with ˙OH playing a secondary role. Based on these intermediates that are produced, the degradation pathway of PFOA can be divided into three routes (as shown in Fig. 16): route 1, proton (H^+^) abstraction from perfluorooctanoic acid (PFOA) leads to the formation of C_7_F_15_COO˙, which subsequently undergoes the Photo-Kolbe decarboxylation reaction to generate 
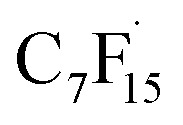
, a perfluoroalkyl radical. The radical reacts with molecular oxygen (O_2_) through a series of oxidative reactions to produce perfluoroheptanoic acid (C_6_F_13_COOH). In route 2, under the assistance of h^+^, ˙O_2_^−^ undergoes nucleophilic substitution (S_N_2) on the α-C of PFOA, causing the cleavage of one F^−^ on α-C. This reaction is followed by the Photo-Kolbe reaction, which results in decarboxylation and the formation of C_6_F_13_CHF(OH). After dehydration, the corresponding fully fluorinated heptaldehyde, C_6_F_13_CHO, is produced. Finally, under the activity of ˙OH and ˙O_2_^−^, heptaldehyde is oxidized to the corresponding perfluorocarboxylic acid, C_6_F_13_COOH. In route 3, based on the replacement of the second pathway *via* the generation of C_6_F_13_CFO_2_HCOO^−^ by F^−^, followed by substitution of another F^−^, the corresponding perfluorooctanoic acid C_6_F_13_COCOOH is produced after dehydration, and then it is decarboxylated by Photo-Kolbe reaction to form the corresponding perfluorooctanal C_6_F_13_CHO. Finally, under the action of ˙OH and ˙O_2_^−^, it is oxidized to the corresponding carboxylic acid C_6_F_13_COOH.

## 4. Conclusion

In this study, In_2_O_3_ was prepared from indium nitrate and the In_2_O_3_/BiOCl p–n heterojunction was synthesized by combining it and BiOCl. The preparation process and photodegradation process of In_2_O_3_/BiOCl were investigated using the defluorination efficiency of PFOA under UV illumination as a performance indicator. The results showed that when the prepared In_2_O_3_/BiOCl p–n heterojunction was excited under illumination, the photogenerated electrons on the conduction band of BiOCl would transfer to the CB of In_2_O_3_, while holes would still remain on the VB of BiOCl, resulting in effective separation of photogenerated electron–hole pairs, thereby enhancing its catalytic activity. Moreover, the prepared heterojunction exhibits less sensitivity to pH and demonstrates excellent degradation activity for PFOA in the wider range of acidic and alkaline environments (up to pH ≤ 10).

The photocatalyst that was prepared under the condition of *m*(In_2_O_3_) : *m*(BiOCl) = 3 : 7 and calcined at 300 °C exhibits the highest efficiency in PFOA defluorination. At a catalyst dosage of 0.2 g L^−1^, degradation solution pH value of 5.00, and PFOA concentration of 20 mg L^−1^, the defluorination rate of PFOA reached 84.01%. Even after the catalyst was used four times, the defluorination efficiency of PFOA still exceeded 76.44%, which demonstrated its excellent stability.

In the photodegradation process of PFOA by the In_2_O_3_/BiOCl p–n heterojunction, h^+^ and ˙O_2_^−^ play a primary role, while ˙OH plays a minor role in the defluorination process of PFOA. The degradation of PFOA is aided by these two components of In_2_O_3_ and BiOCl in the heterojunction. PFOA molecules are adsorbed on BiOCl with plentiful oxygen vacancies and then gradually lose CF_2_ units under the action of ˙O_2_^−^ produced by the CB reduction of In_2_O_3_, and are ultimately degraded into H_2_O and CO_2_.

## Data availability

The data supporting this article have been included as part of the ESI.†

## Conflicts of interest

There are no conflicts to declare.

## Supplementary Material

RA-015-D5RA01317H-s001
