# Supplementary figures and images for: Study on the performance and mechanism of a p–n type In2O3/BiOCl heterojunction prepared using a sacrificial MOF framework for the degradation of PFOA
Source: RSC Adv. 2025 May 8;15(19):15029–51. doi: 10.1039/d5ra01317h (PMC12060136; doi:10.1039/d5ra01317h)

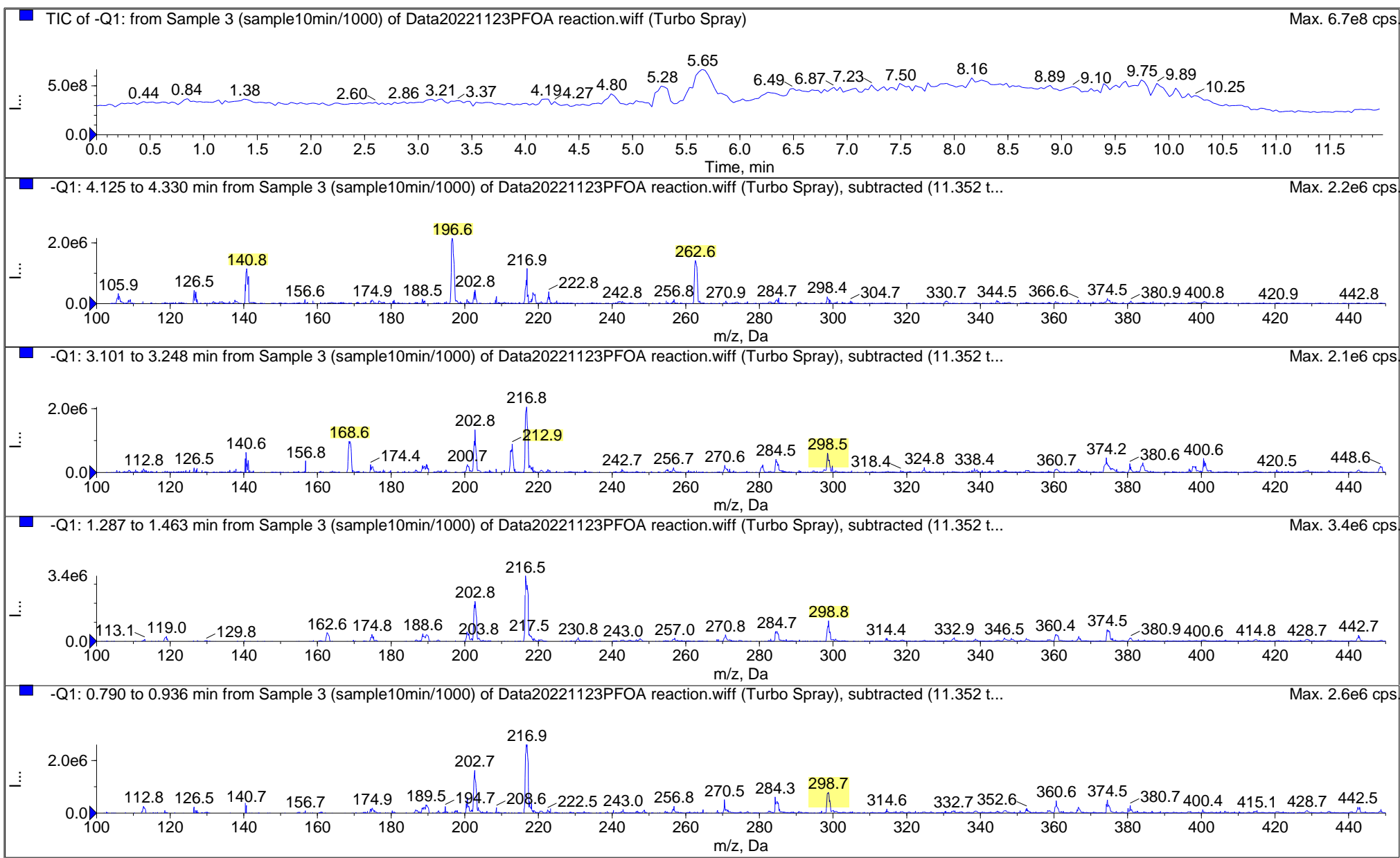

Supplement: RA-015-D5RA01317H-s001 [file RA-015-D5RA01317H-s001.zip › Supplementary Information/LC-MS/20221123-10min-1.pdf]

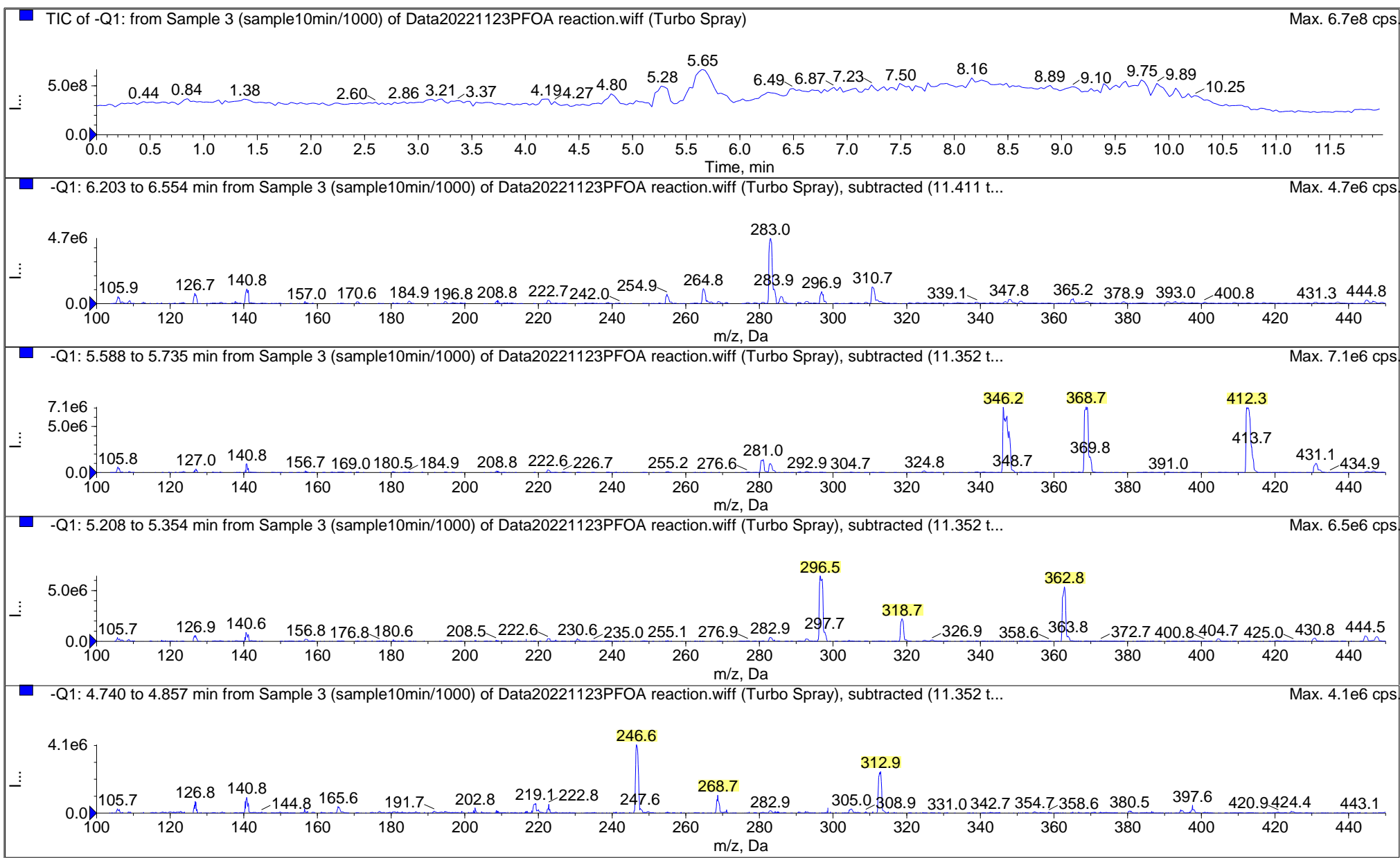

Supplement: RA-015-D5RA01317H-s001 [file RA-015-D5RA01317H-s001.zip › Supplementary Information/LC-MS/20221123-10min-2.pdf]

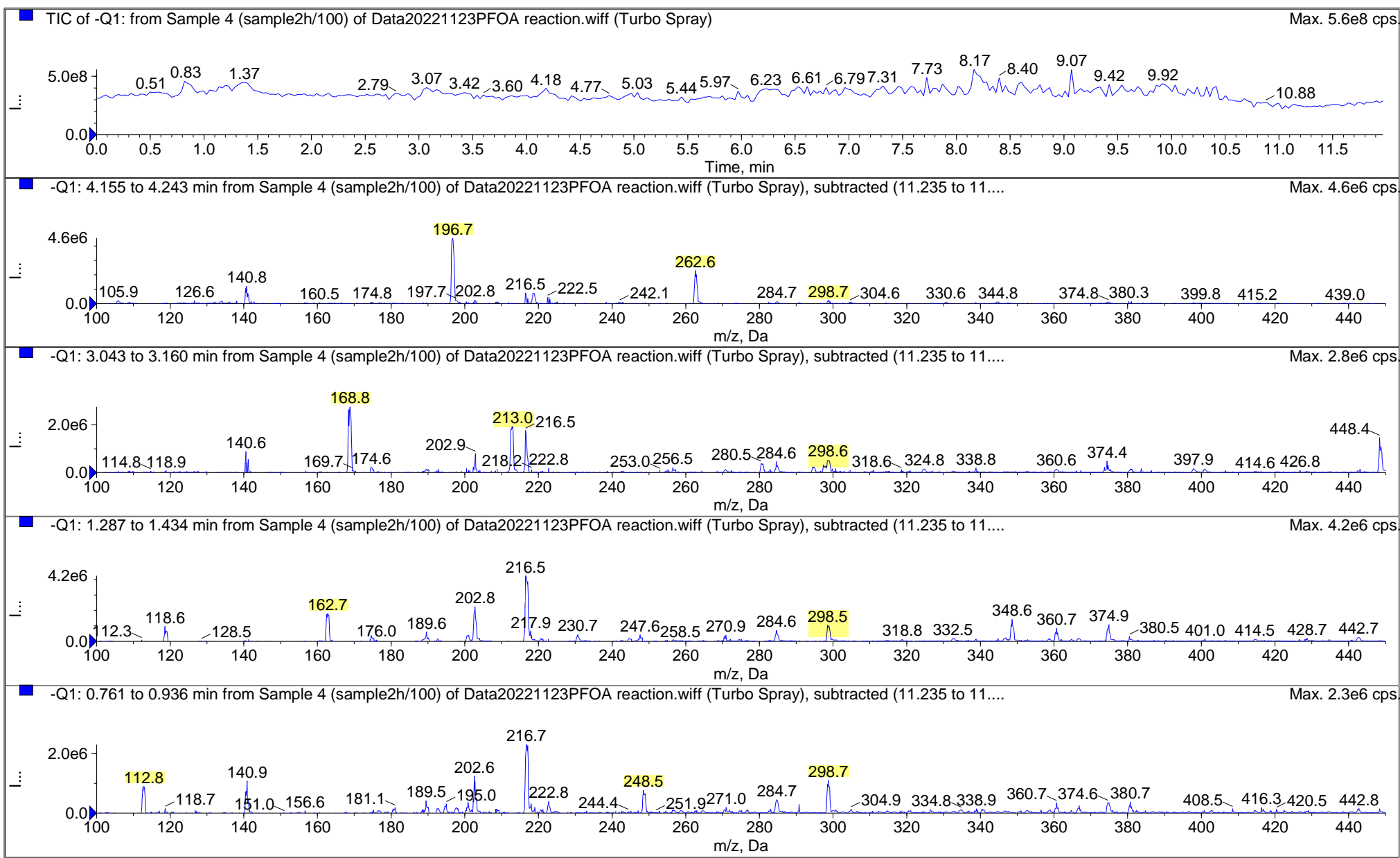

Supplement: RA-015-D5RA01317H-s001 [file RA-015-D5RA01317H-s001.zip › Supplementary Information/LC-MS/20221123-2h-1.pdf]

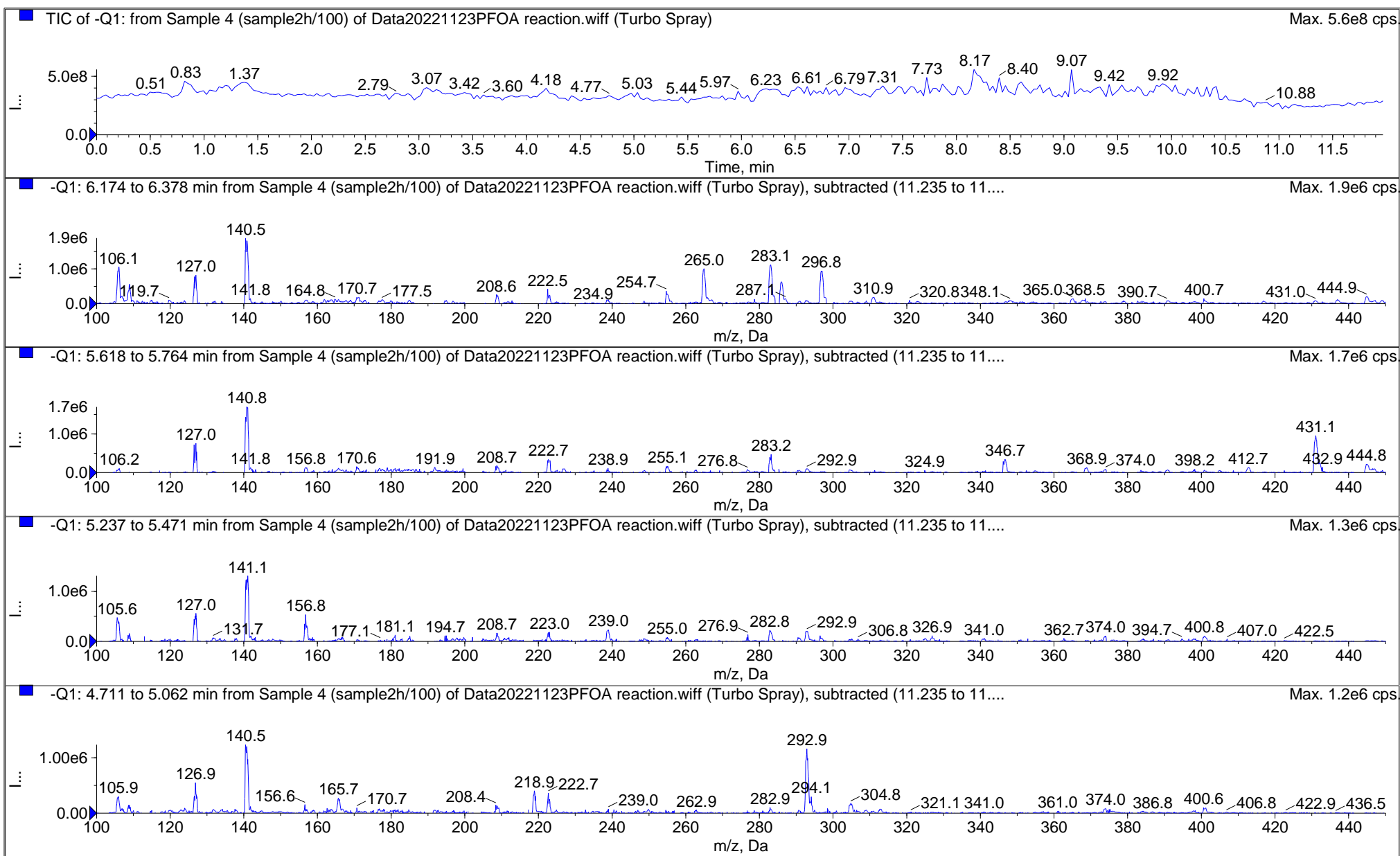

Supplement: RA-015-D5RA01317H-s001 [file RA-015-D5RA01317H-s001.zip › Supplementary Information/LC-MS/20221123-2h-2.pdf]

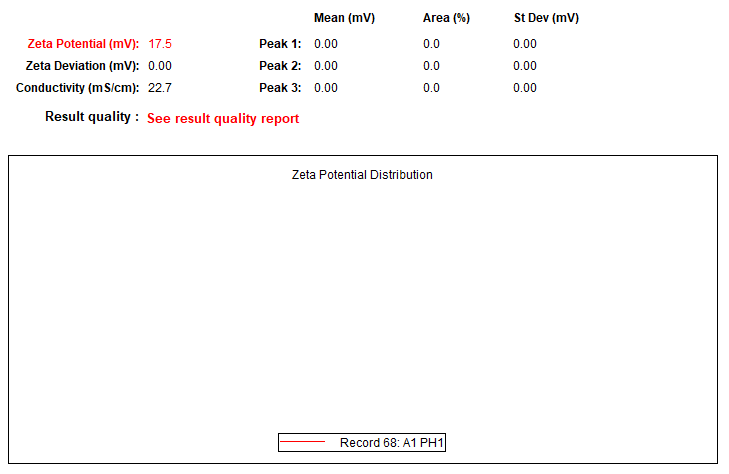

Supplement: RA-015-D5RA01317H-s001 [file RA-015-D5RA01317H-s001.zip › Supplementary Information/Testing of catalyst surface charge at different pH values/A1 PH1 1.png]

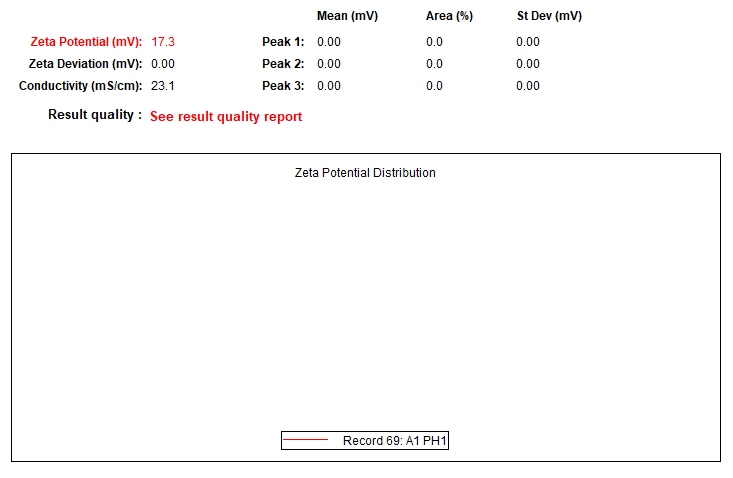

Supplement: RA-015-D5RA01317H-s001 [file RA-015-D5RA01317H-s001.zip › Supplementary Information/Testing of catalyst surface charge at different pH values/A1 PH1 2.png]

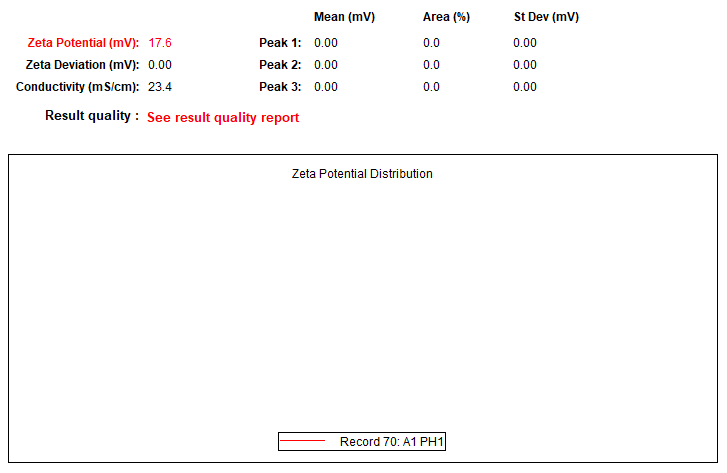

Supplement: RA-015-D5RA01317H-s001 [file RA-015-D5RA01317H-s001.zip › Supplementary Information/Testing of catalyst surface charge at different pH values/A1 PH1 3.png]

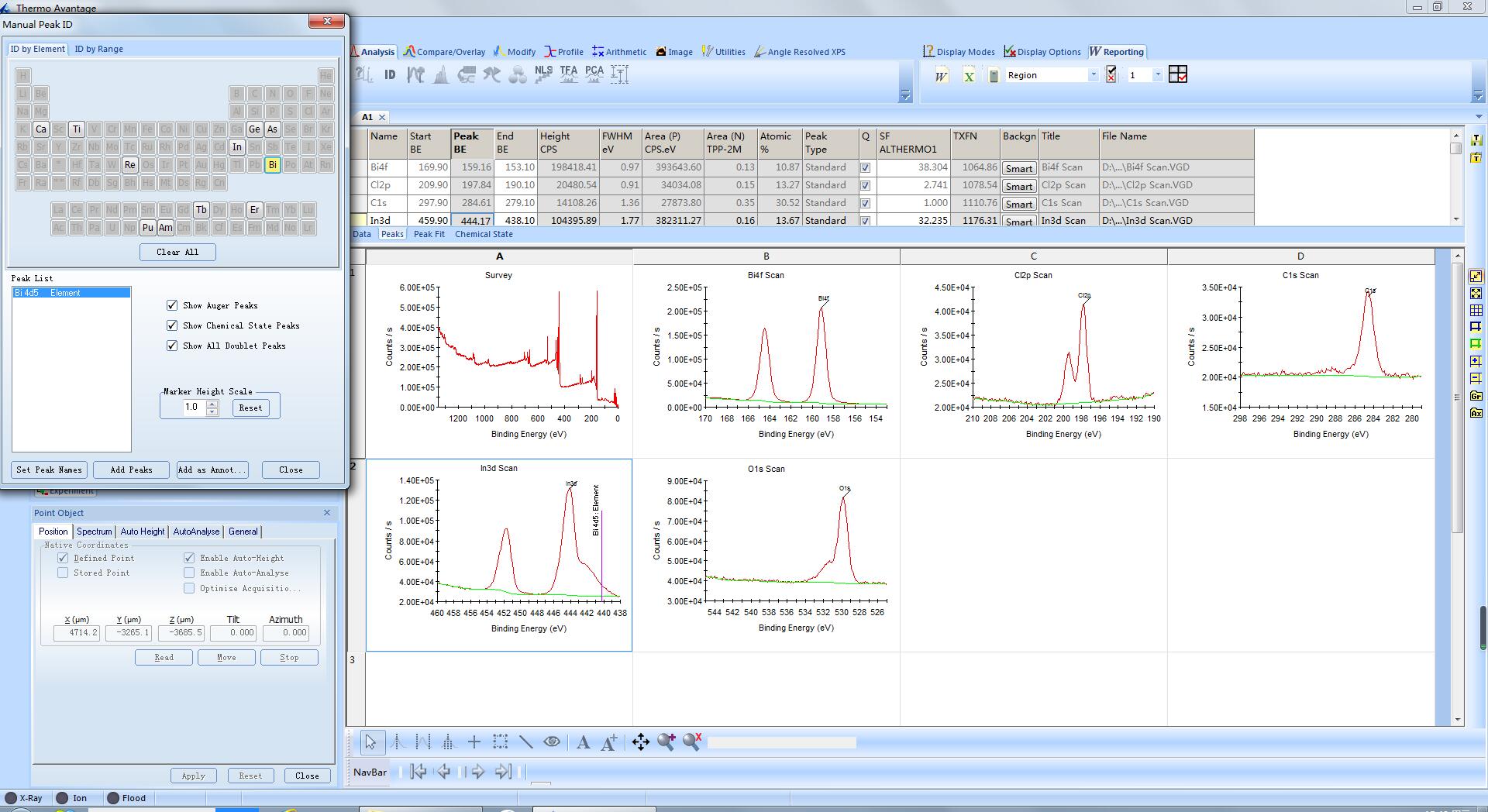

Supplement: RA-015-D5RA01317H-s001 [file RA-015-D5RA01317H-s001.zip › Supplementary Information/XPS-data/QQ screenshot.jpg]
